# Design, synthesis, and evaluation of peptide‐imidazo[1,2‐*a*]pyrazine bioconjugates as potential bivalent inhibitors of the VirB11 ATPase HP0525

**DOI:** 10.1002/psc.3353

**Published:** 2021-06-17

**Authors:** James R. Sayer, Karin Walldén, Hans Koss, Helen Allan, Tina Daviter, Paul J. Gane, Gabriel Waksman, Alethea B. Tabor

**Affiliations:** ^1^ Department of Chemistry UCL London UK; ^2^ MedPharm Ltd Guildford UK; ^3^ Institute of Structural and Molecular Biology UCL London UK; ^4^ Department Biochemistry and Biophysics Stockholm University Solna Sweden; ^5^ Department of Biochemistry and Molecular Biophysics Columbia University New York New York USA; ^6^ Department of Biological Sciences ISMB Biophysics Centre London UK; ^7^ The Institute of Cancer Research London UK; ^8^ Wolfson Institute for Biomedical Research UCL London UK; ^9^ Abcam Cambridge UK; ^10^ Present address: MedPharm Ltd, R&D Centre Unit 3/Chancellor Court, 50 Occam Road, Surrey Research Park Guildford GU2 7AB UK; ^11^ Present address: Department Biochemistry and Biophysics Stockholm University Tomtebodavägen 23A Solna SE‐171 65 Sweden; ^12^ Present address: Department of Biochemistry and Molecular Biophysics Columbia University 701 West 168th Street New York NY 10032 US; ^13^ Present address: The Institute of Cancer Research 237 Fulham Road London SW3 6JB UK; ^14^ Present address: Abcam, Discovery Drive Biomedical Campus Cambridge CB2 0AX UK

**Keywords:** antimicrobial, ATPase inhibitor, bivalent inhibitor, docking, protein–protein interaction (PPI)

## Abstract

*Helicobacter pylori* ( *H. pylori* ) infections have been implicated in the development of gastric ulcers and various cancers: however, the success of current therapies is compromised by rising antibiotic resistance. The virulence and pathogenicity of *H. pylori* is mediated by the type IV secretion system (T4SS), a multiprotein macromolecular nanomachine that transfers toxic bacterial factors and plasmid DNA between bacterial cells, thus contributing to the spread of antibiotic resistance. A key component of the T4SS is the VirB11 ATPase HP0525, which is a hexameric protein assembly. We have previously reported the design and synthesis of a series of novel 8‐amino imidazo[1,2*‐a*]pyrazine derivatives as inhibitors of HP0525. In order to improve their selectivity, and potentially develop these compounds as tools for probing the assembly of the HP0525 hexamer, we have explored the design and synthesis of potential bivalent inhibitors. We used the structural details of the subunit–subunit interactions within the HP0525 hexamer to design peptide recognition moieties of the subunit interface. Different methods (cross metathesis, click chemistry, and cysteine‐malemide) for bioconjugation to selected 8‐amino imidazo[1,2*‐a*]pyrazines were explored, as well as peptides spanning larger or smaller regions of the interface. The IC_50_ values of the resulting linker‐8‐amino imidazo[1,2*‐a*]pyrazine derivatives, and the bivalent inhibitors, were related to docking studies with the HP0525 crystal structure and to molecular dynamics simulations of the peptide recognition moieties.

## Graphical abstract

The virulence and pathogenicity of *H. pylori* are mediated by the type IV secretion system (T4SS). A key component of the T4SS is the hexameric protein assembly VirB11 ATPase HP0525, and small molecule inhibitors of HP0525 have been previously reported. In order to improve their selectivity, and potentially develop these compounds as tools for probing the assembly of the HP0525 hexamer, we report the design, synthesis, and evaluation of potential bivalent inhibitors, using the subunit‐subunit interactions within the HP0525 hexamer to design peptide recognition moieties of the subunit interface.

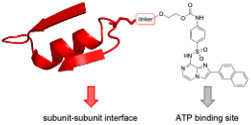

## 1 INTRODUCTION

Since its isolation in 1983,^1^ *Helicobacter pylori* (*H. pylori*) has been identified as the most common human bacterial infection, present in approximately half of the world's population.^2^ This type of Gram‐negative bacteria is found in the human stomach and causes illnesses such as gastric ulcers, gastritis, and various cancers including mucosa‐associated lymphoid tissue (MALT)‐lymphoma and gastric adenocarcinoma.^3^, ^4^ Although the majority of those infected are asymptomatic, *H. pylori*‐positive patients have a 10%–20% lifetime risk of developing ulcer disease and a 1%–2% risk of developing gastric cancer,^5^ and as such, the bacteria have been classified as a category 1 carcinogen.^6^ It has been estimated that between 2008 and 2015, the proportion of noncardiac gastric cancer attributable to *H. pylori* increased from 74.7% to 89.0%.^7^ The current standard treatment of *H. pylori* infections is based upon triple therapy,^8^, ^9^ consisting of a proton pump inhibitor and a choice of two antibiotics or quadruple therapy^8^, ^9^, ^10^ in which bismuth compounds are also used. The success of these therapies is unfortunately under pressure due to rising antibiotic resistance and off‐target effects caused by prolonged antibiotic treatment.^11^ Multi‐drug resistant *H. pylori* (resistance to ≥3 antibiotics of different classes) ranges from ≤10% in Europe to >20% in India and >40% in Peru.^12^ So far, no eradication therapy can provide high eradication rates (>90%), and a variety of approaches to targeting *H. pylori* including antivirulence therapeutics, mucolytic agents, and antibacterial agents are currently being investigated.^13^

Gram‐negative bacteria have evolved a range of secretion systems to transport substrates across their cell membranes. They can release small molecules, proteins, and DNA into extracellular space or can inject these substrates into a target cell.^14^ Seven classes of double membrane‐spanning secretion systems, Type I–Type VII, have so far been identified. The Type IV secretion system (T4SS) is of particular interest, as it mediates the transfer of plasmid DNA between bacterial cells, thus contributing to the spread of antibiotic resistance genes. Inhibitors of the T4SS are therefore of interest as antimicrobial agents with the potential to slow the development of antimicrobial resistance.^15^

**FIGURE 1:**
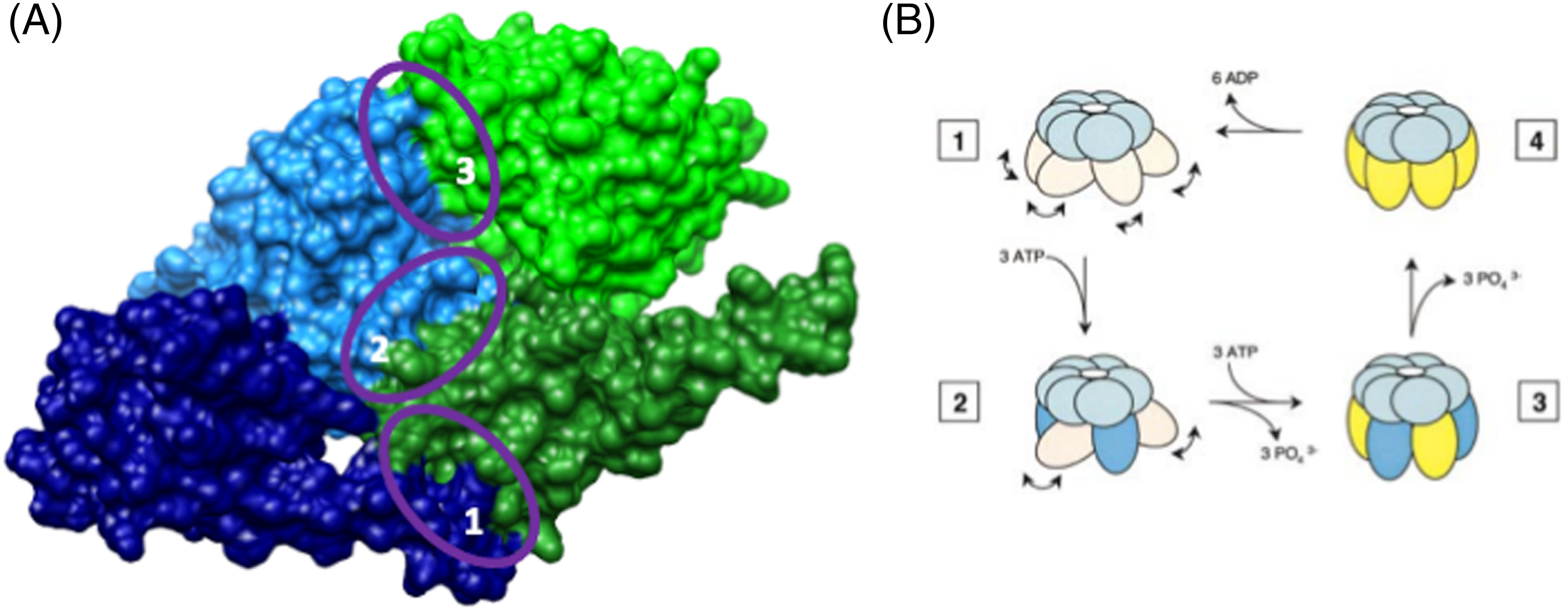
(A) Illustration of the three major contacts in the VirB11 ATPase HP0525 subunit–subunit interface. Blue: Subunit A (dark blue = NTD, light blue = CTD), green: Subunit B (dark green = NTD, light green = CTD); 1: NTD‐NTD interactions, 2: NTD‐CTD interactions, 3: CTD‐CTD interactions. Image generated using chimera.^25^ (B)4hematic representation for the mode of action of VirB11 ATPases. NTD (light pink), CTD (light blue). ATP binding (blue) and ADP (yellow). In **1** (nucleotide‐free form), the CTD maintains the pseudo‐scaffold and the NTD is mobile. Binding of three ATP molecules then locks three of the subunits into a rigid conformation (state **2**). Hydrolysis of the first three ATPs to ADP together with binding of a further three ATP molecules to the remaining subunits leads to state **3**. Hydrolysis of the final ATP molecules leads to a rigid hexamer (state **4**). Finally, release of the six ADPs allows the hexamer to revert to its nucleotide‐free asymmetric form (state **1**). Reproduced with permission (*The EMBO Journal*) from Savvides et al.^24^

*H. pylori* can be grouped into two classes,^16^, ^17^ and the more virulent type I strains contain the cytotoxin‐associated genes pathogenicity island (*cag*PAI)^18^ and are referred to as CagA^+^ strains. The *cag*PAI consists of 31 genes, the majority of which code for T4SS,^5^, ^16^ which in *H. pylori* is responsible for penetrating the gastric epithelial cells and facilitating the translocation of toxic bacterial factors into host cells.^19^, ^20^, ^21^ T4SS are multifunctional macromolecular nanomachines, incorporating 12 different types of protein subunit with specific roles in the complex.^22^ The VirB11 ATPase HP0525 is a key component of this complex, which provides energy to power the system and is required to drive CagA secretion and delivery. It is also hypothesized to act as a molecular switch that controls the export of DNA and the assembly of the T4SS pilus. Hence, there has been much interest in the structure and function of this protein. There have been extensive studies into the crystal structures of the *H. pylori* VirB11 homolog HP0525 bound to both ADP^23^ and the non‐hydrolysable ATPγS as well as the nucleotide‐free, *apo*‐form.^24^ HP0525 forms double hexameric ring structures where each subunit monomer consists of 328 amino acid residues comprising the *N*‐terminal domain (NTD) and *C*‐terminal domain (CTD). Each of the NTDs and CTDs form two separate ring structures (Figure 1a) with the CTD ring forming a six‐clawed grapple mounted on the hexameric ring formed by the NTD creating a cylindrical chamber. The structures of ADP‐ and ATPγS‐HP0525 are virtually identical; however, *apo*‐HP0525 exists as an asymmetric hexamer, very different to that of the nucleotide bound forms. Examination of the crystal structures of HP0525 showed that nucleotide binding and not hydrolysis is responsible for ATP‐induced conformational changes^23^, ^24^ and allowed a mechanism for the mode of action of the ATPase to be proposed (Figure 1b).

Selective inhibitors of VirB11 have the potential to combat the proteins and pathways that lead to pathogenic, symptomatic colonization of the stomach, and may also be useful chemical biology tools to elucidate the pathways of assembly of the T4SS macromolecular complex. However, only a small number of small molecule inhibitors of *H. pylori* HP0525 have so far been reported.^15^ Thiadiazolidine‐3,5‐diones^26^ and the non‐competitive inhibitor 4‐(5‐methylpyridin‐2‐yl)oxybenzoic acid^27^ have been shown to inhibit VirB11 ATPase, whereas heterocyclic 2‐pyridone inhibitors^28^ have been shown to disrupt T4SS apparatus biogenesis by attenuating the delivery of peptidoglycan and CagA to host cells. Unsaturated 2‐alkynoic fatty acids have also been shown to inhibit conjugation in TrwD, a VirB11 homolog.^29^

We have previously reported the synthesis and high throughput screen of a series of novel 8‐amino imidazo[1,2*‐a*]pyrazine derivatives against VirB11 ATPase HP0525.^30^ Biochemical evaluation showed moderate to good potency, highlighting these compounds as competitive inhibitors of HP0525 and potential antibacterial agents. However, as these molecules probably bind at the ATPase active site, to avoid inhibiting other ATPases in mammalian cells, we wished to try to improve their selectivity for VirB11 ATPase. We were also motivated to exploit the potential of these compounds as chemical biology tools for probing the assembly of the HP0525 hexamer. In order to achieve both of these aims, in this paper, we have explored the design and synthesis of potential bivalent inhibitors of the VirB11 ATPase HP0525.

Affinity and specificity of an inhibitor can be greatly enhanced by taking advantage of hydrophobic/hydrophilic features near the enzyme active site. By linking an active site binding compound to a moiety that interacts with these features on the enzyme surface, it is possible to differentiate among enzymes. The resulting compounds are known as bivalent inhibitors. Bivalent molecules comprising a small molecule and a peptide have been frequently studied, as this approach has been particularly valuable in designing selective kinase inhibitors,^31^ in which an ATP‐binding site directed small molecule is tethered to a protein substrate site of the protein kinase. Examples of potent and selective bivalent inhibitors include ATPγS‐IRS727 peptide bioconjugates as insulin receptor protein tyrosine kinase (IRK) inhibitors^32^ and high‐affinity nonselective kinase inhibitor K252a‐ protein kinase inhibitor protein (PKI) bioconjugates as inhibitors of protein kinase A (PKA).^33^ Bivalent inhibitors can also be designed to selectively disrupt protein–protein interactions (PPIs).^34^

In this work, we have used the imidazo[1,2‐*a*]pyrazine compounds previously synthesized to target the ATP binding site of HP0525, whereas a peptide sequence has been rationally designed to disrupt the peptide–peptide interactions of the subunit–subunit interface and, in doing so, disrupting hexamer formation.

## 2 MATERIALS AND METHODS

### 2.1 General methods, reagents, and chemical synthesis

General methods for chemical synthesis are included in the supporting information. All chemicals were of commercial quality and have been used without additional purification; **19**,^35^ **20**,^36^ **21**,^37^ and **25** ^30^ were synthesized according to literature procedures. The synthetic procedures, purification methods, and compound characterizations of all other compounds are reported in the supporting information.^38^

### 2.2 Solid phase peptide synthesis

Amino acids and resins for peptide synthesis were purchased from Novobiochem, UK. HPLC grade solvents for peptide synthesis and HPLC purification were purchased from Sigma, UK and VWR. All peptides were synthesized on a MultiSynTech Syro I automated system. Either pre‐loaded Fmoc‐Asp (O^*t*^Bu)‐NovaSyn® TGT resin (0.200 mmol/g, 100 mg, 20.0 μmol) or pre‐loaded Fmoc‐Leu‐NovaSyn® TGT resin (0.210 mmol/g, 100 mg, 21.0 μmol) were used in all cases. All resins were pre‐swelled in DMF for at least 30 min prior to synthesis start. Standard Fmoc solid phase peptide synthesis (SPPS) was employed. The total volume of all reagents in each step was 1.5 ml. All reagents were dissolved in HPLC grade DMF.

#### 2.2.1 Fmoc removal

The reaction syringe containing *N*‐terminal Fmoc‐protected peptide was added piperidine in DMF (40% v/v, 1.5 ml). The mixture was agitated for 20 s every min for a total of 3 min. The reagents were removed by filtration under vacuum, and the resin washed with DMF (4 × 1.5 ml). Piperidine in DMF solution (40% v/v, 0.75 ml) was added to the reaction syringe followed by DMF (0.75 ml) to make an overall 20% v/v solution of piperidine in DMF. This mixture was agitated for 20 s every min for a total of 10 min. The reagents were removed by filtration under vacuum, and the resin washed with DMF (6 × 1.5 ml).

#### 2.2.2 Amino acid coupling

The reaction syringe was added Fmoc‐protected amino acid (0.600 ml, 0.140 M in DMF, 4 eq.), HBTU (0.600 ml, 0.140 M in DMF, 4 eq.), and DIPEA (0.300 ml, 0.560 M in DMF, 8 eq.). The mixture was agitated for 20 s every 3 min for a total of 40 min. The reagents were removed by filtration under vacuum, and the resin washed with DMF (4 × 1.5 ml).

#### 2.2.3 Peptide cleavage and side chain deprotection

The resin was washed with CH_2_Cl_2_ (3 × 3 ml), MeOH (3 × 3 ml) and Et_2_O (3 × 3 ml) and dried (desiccator) followed by adding TFA/EDT/H_2_O/TIPS (88:5:4:2; 3 ml) to the reaction syringe. The syringe was then agitated for 3 h at RT. The cleavage cocktail was drained from the vessel under vacuum and Et_2_O (~10–15 ml) added to the filtrate. The resultant precipitate in solution was stored at −20°C for 30 min prior to being spun at 4000 rpm for 10 min at 4°C to produce a crude peptide pellet. The supernatant Et_2_O was decanted off, and the peptide washed a further three times with Et_2_O. The crude peptide pellet was then re‐dissolved in minimum water and freeze‐dried for storage prior to purification.

#### 2.2.4 General peptide purification

The peptides were analyzed and purified via reverse phase HPLC using a Varian ProStar system with a Model 210 solvent delivery module and a Model 320 UV detector. The preparative purification was performed using either a Discovery®BIO Wide Pore C18 (Varian; 100 × 21.2 mm, 5 μm beads, flow rate of 10 ml/min), Discovery®BIO Wide Pore C18 (Varian; 25 cm × 21.2 mm, 10 μm beads, flow rate of 8 ml/min) or an Onyx Monolithic Semi‐Prep C18 (Phenomenex®; 100 × 10 mm, 2 μm macropore size, 13 nm mesopore size, flow rate 10 ml/min), loaded with 200–400 μl aliquots of a 10–20 mg/ml solution of peptide dissolved in 0.1% TFA containing H_2_O. The mobile phase was a decreasing gradient of H_2_O/0.1% TFA (A) in MeCN/0.1% TFA (B). Precise gradients are reported for each peptide. The fractions containing the correct peak were pooled, and the solvent was removed under reduced pressure to approximately 2 ml, and the solution freeze‐dried.

The purified peptide was analyzed by analytical HPLC using an Discovery®BIO Wide Pore C18 (Varian; 25 cm × 4.6 mm, 10 μm beads, flow rate of 1 ml/min) or Onyx monolithic C18 column (Phenomenex®; 100 × 3.0 mm, 2 μm macropore size, 13 nm mesopore size, flow rate 1.0 ml/min). Precise gradients reported for each peptide. The analysis of the chromatograms was conducted using Star Chromatography Workstation software Version 1.9.3.2. ES‐MS analysis was performed on a Waters Acquity Ultra Performance LC/MS machine. MALDI analysis was carried out using a Waters MALDI MICRO MX, Micromass Technologies.

Analytical HPLC traces, ES+, and MALDI spectra of peptides **1**–**9** can be found in the supporting information.

### 2.3 Conjugation

#### 2.3.1 Cysteine‐maleimide conjugation (solution)

The purified peptide was taken up in DMF (approximately 2 mg/ml). An aliquot of a stock solution of **17** in DMF (2.87 mM) was added such that the volume added corresponded to 3 eq, and the reaction mixture stirred at RT for 3 h. The solvent was removed in vacuo and the crude material purified via reverse phase preparative HPLC.

#### 2.3.2 “Click” chemistry (on resin)

The peptides were synthesized according to standard SPPS, with all side chain and the terminal Fmoc protecting groups left on. The peptide was also left bound to the resin, and all subsequent reactions were carried out in the syringe; **164** (43.3 mg, 0.080 mmol, 4 eq) in DMF (2 ml) was added to the reaction syringe followed by the addition of a suspension of CuI (76.0 mg, 20 eq) and sodium ascorbate (158 mg, 40 eq) in H_2_O/^*t*^BuOH (2:1; 750 μl) to give a total solvent composition of DMF/H_2_O/^*t*^BuOH (8:2:1). The mixture was agitated at RT for 16 h, followed by evacuating the solvent and washing with DMF (3 × 3 ml), MeOH (2 × 3 ml), CH_2_Cl_2_ (3 ml), and DMF (2 × 3 ml). The terminal Fmoc was removed, followed by cleavage of the peptide from the resin and deprotection of the side‐chain protecting groups using conditions stated above.

#### 2.3.3 Cysteine‐maleimide conjugation (on resin)

The peptides were synthesized according to standard SPPS, with all side chain and the terminal Fmoc protecting groups left on. The peptide was also left bound to the resin, and all subsequent reactions were carried out in the syringe. The S^*t*^Bu protecting group on R240C was selectively deprotected by: washing the resin‐bound peptide with CH_2_Cl_2_ (3 × 3 ml); soaking in EtOH/CH_2_Cl_2_/H_2_O (4:6:1; 3 ml); purging with Ar; adding nBu_3_P (50.0 μl, 0.200 mmol, 10 eq); and agitating for 3 h at RT. The syringe was evacuated and washed with CH_2_Cl_2_ (2 × 3 ml), MeOH (2 × 3 ml), CH_2_Cl_2_ (2 × 3 ml), and DMF (2 × 3 ml). Maleimide conjugation to the free thiol in R240C was carried out by adding **5** (42.0 mg, 0.060 mmol, 3 eq) in DMF (3 ml) and agitating at RT for 16 h. The syringe was evacuated and washed with DMF (3 × 3 ml). The terminal Fmoc was removed, followed by cleavage of the peptide from the resin and deprotection of the side‐chain protecting groups using conditions stated above.

Analytical HPLC traces, ES+, and MALDI spectra of conjugated peptides **28**, **29**, and **30** can be found in the supporting information.

### 2.4 Biological assays

The HP0525 protein was produced in the *E. coli* strain BL21 Star (DE3) (Invitrogen) as described previously.^39^ The protein concentration was estimated spectroscopically using a NanoDrop (Thermo Scientific) and a calculated extinction coefficient at 280 nm, based on the amino acid composition. The ATPase activity of HP0525 was measured, with and without a specific amount of compound present, using an in vitro ATPase colorimetric assay kit (Innova Biosciences). The assay was performed in 96‐well ELISA microplates (Greiner Bio‐One), using a multipipette/robot.

A volume of 49 μl of a substrate/buffer solution (200 mM tris(hydroxymethyl)aminomethane [TRIS], pH 7.5; 5 mM MgCl_2_; 250 μM ATP; and 10% DMSO) was added to each assigned well, followed by the addition of 1 μl of compound (at 0.5; 5 or 50 mM to achieve the final concentrations of 5; 50 and 500 μM in the reaction, respectively) in DMSO (or 1 μl DMSO to controls). The solutions were mixed carefully by pipetting. The reaction was started by the addition of 50 μl of 0.106 μM HP0525 to each well (except the negative control, see text below), and the reaction plate was directly transferred to 37°C for 30 min of incubation. The reaction was stopped by the addition of the gold mix according to the standard protocol of the kit. The absorbance at 620 nm was measured after 30 min at RT. For each compound, the percentage of absorbance relative to non‐inhibited HP0525 was calculated, after subtracting the absorbance value of the negative control. In the negative control, the protein was added after the gold mix, as described in the standard protocol of the kit, which when used as a blank corrects for all free P_i_ not produced by the enzyme during the 30 min incubation at 37°C. A known inhibitor of HP0525 (CHIR02)^26^ was used as a control inhibitor. All measurements were made in duplicate.

A selection of the compounds was assayed as above at additional concentrations ranging between 1 and 50 μM (the measuring points were optimized to cover the range to fit a sigmoidal dose response curve, and at the same time ensure compounds were not precipitating) from which IC_50_ values were calculated (Figure S1). Each compound was screened on three separate plates, and each plate was read twice, and a mean was calculated for each concentration. The data were normalized by subtracting the negative control (0% active) and relating it to the positive control (100% active). The software GraphPad Prism 5 was used to generate two dose–response curves (Log [inhibitor] vs. normalized response [standard and variable slope]), and the IC_50_ values were calculated from each.

The same assay format was used for the enzyme kinetics experiments, where the activity of HP0525 was tested at various concentrations of ATP (0–500 μM). The data were plotted and fitted to the Michaelis–Menten equation to obtain V_max_, from which K_m_ can be calculated.

### 2.5 Molecular modelling

AutoDock 4.0^40^ and Vina^41^ were used for the in silico docking of the compounds into .pdb file: 1G6O (ADP‐HP0525). All heteroatoms (H_2_O, PEG, and ADP molecules) were removed from the .pdb file, which was converted into .pdbqt (includes partial charges and autodock atom types) format using AutoDock Tools (ADT), with the addition of polar hydrogens and Kollman charges. AutoDock 4.0 requires the following files: *enzyme*.pdbqt, *ligand*.pdbqt, *GridFile*.gpf (grid parameter file), and *DockingFile*.dpf (docking parameter file). AutoDock Vina requires the *enzyme*.pdbqt, *ligand*.pdbqt, and *configuration*.txt files. ADT was used to generate the *ligand*.pdbqt file from a .pdb or .mol2 input, which was subjected to no initial energy minimizations. When using PyRx (Open Source software with intuitive user interface for docking), the program automatically converts .pdb or .mol2 files to .pdbqt files. The size of the grid box used is as follows: *x* (14 Å), *y* (16 Å), and *z* (24 Å). Coordinates of the grid box used for the docking into sites A (−40.36, 53.652, and 22.3) and B (−12.034, 24.627, and 22.363) of 1G6O. In the case of AutoDock 4.0, the grid file and docking file were generated using ADT. In the case of AutoDock Vina, the coordinates were used in the configuration text file, and a value of 8 was used for the exhaustiveness. Linux was used to run the AutoDock 4.0 program, and either cmd or PyRx was used to run AutoDock Vina. The output file from 4.0 was in .dlg format (docked log files), which can be converted to .pdbqt using ADT and viewed in PyMOL. In order to visualize the docking file using chimera, the file needs to be converted further to the .pdb, using PyMOL. Vina exports the docking files as .pdbqt.

MOE 2009 was used for evaluation of the bifunctional reagents.^42^ The R240 residue was mutated to the relevant residue, and the molecule was built using the “Build Molecule” function. For example, select residue, add H, convert to C, add H, convert to C, add H, and so forth.

The software package GROMACS v. 4.5.4 was used for molecular dynamics simulations.^43^ Three peptide fragments from the HP0525 crystal structure^24^ 1NLZ were extracted as .pdb structure files:

1. αF: subunit F ‐ residues 229–242
2. αF‐β10 subunit F ‐ residues 229–246
3. β9‐αF‐β10 subunit F ‐ residues 218–246

The dynamics of these peptides were simulated in the following way: the peptide structures were converted into a model with an AMBER99SB‐ILDN43 forcefield. The peptide was then placed in a 40 Å × 40 Å × 40 Å box. After energy minimization (steepest descent, dt = 0.002 ps, 20,000 steps), the system was filled with SPC‐water. Water molecules were recursively substituted by NaCl until c (NaCl) = 0.15 M. The system was minimized again using the same conditions as mentioned above. The actual atomistic molecular dynamics simulation of this system was performed on a desktop computer with two cores using the following conditions: duration = 10 ns; dt = 0.002 ps; temperature coupling: Berendsen thermostat, T = 300 K, τT = 0.1 ps; pressure coupling: isotropic Parinello–Rahman, p = 1.0 bar, τT = 1.0 ps, compressibility = 4.6·10–5 bar‐1. The system had 6494/6492/6463 elements in αF/αFβ10/β9‐αF‐β10. The resulting trajectory was progressively fit to the first (centered) frame. VMD44 was used for visualization and for calculating Ramachandran plots.

### 2.6 Circular dichroism spectroscopy

The experiments were performed at the ISMB Biophysics Centre. Circular dichroism spectra were measured with a Jasco J‐720 spectropolarimeter. Three different peptides were weighted first: αF‐loop **1** 0.5 ± 0.1 mg 14 residues, MW/residue = 113.9 g/mol; αF‐β10 **2** 0.4 ± 0.1 mg 18 residues, MW/residue = 116.1 g/mol; β9‐αF‐β10 **3** 1.2 ± 0.1 mg 29 residues, MW/residue = 114.9 g/mol. The peptides were dissolved in water; their CD spectra were recorded thrice at 1 mg/ml (β9‐αF‐β10 **3** at 0.5 mg/ml) in a Quartz Suprasil cell (pathlength: 0.1 mm). Data were processed with CDTool45 and Dichroweb46. Outliers were excluded, spectra zeroed, averaged, and converted from millidegree units into Δε units, which is a molar unit; the weighed amount of the sample is important for this conversion. Given the weighting errors stated above, data processing was performed for the corresponding range of different sample concentrations. Different algorithms for analysis of the results were tested in Dichroweb.^44^ CONTIN was chosen as the data analysis algorithm. SP175_S3 was chosen as a reference set (others were tested before making this choice). Given α‐helical content was calculated for different protein concentrations, the results are provided as a range.

## 3 RESULTS AND DISCUSSION

### 3.1 Bivalent inhibitor design

To design bivalent inhibitors that would be selective for the VirB11 ATPase, we sought to combine the structural insights gained from the PPIs between the subunits of the hexameric HP0525 complex^24^ with the active site‐directed small molecule inhibitors previously reported.

**FIGURE 2:**
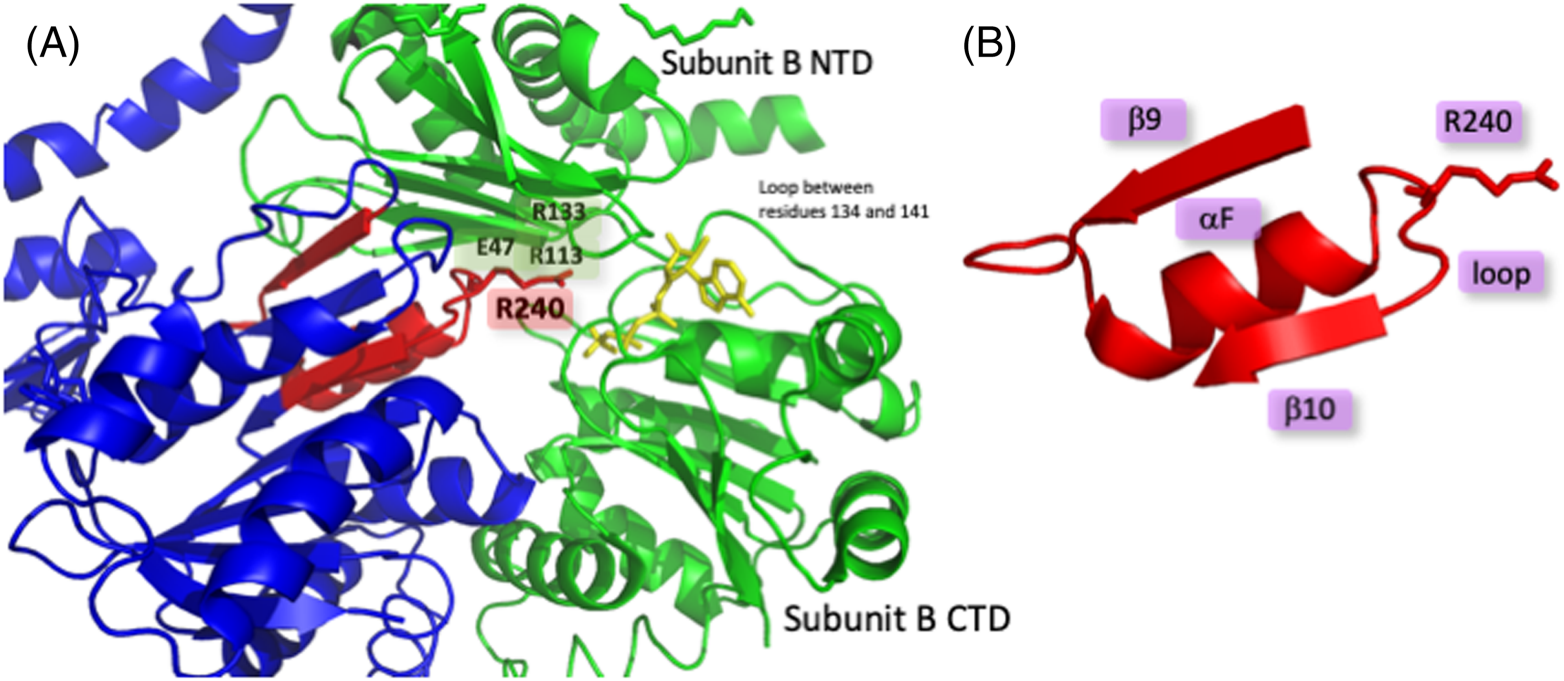
(A) Crystal structure of ATPγS bound to HP0525 (1NLY), highlighting the targeted peptide region at the subunit–subunit interface. ATPγS is in yellow.^24^ (B) Structure of the β9‐αF‐β10 peptide region showing the different secondary structures and the potential first point of attachment for conjugation. Image generated using PyMOL^45^

The HP0525 monomer consists of two domains: the NTD from residues 6 to 136, and the CTD from residues 137 to 328, linked via a short loop between residues 134 and 141 (Figure 2a). The nucleotide binding site is located between the NTD and CTD, with residues from both domains contributing to the binding site. In the hexameric structure of HP0525, each subunit interacts with two flanking subunits, and NTD‐CTD interactions make up the majority of subunit–subunit interfaces. Most of the solvent accessible surfaces of the β‐sheet of the NTD make contact with residues of the β9 sheet, αF helix, αF‐β10 loop, and αE‐β8 loop of the CTD (Figure 2a). There is also a cluster of acidic and basic residues within the NTD‐CTD interface that plays a pivotal role in maintaining the hexameric structure of HP0525. Glu47 from the NTD makes ionic interactions with Arg238 from the adjacent αF‐β10 loop in the CTD. However, Arg113 and Arg133 from the NTD and Arg240 from the αF‐β10 loop in the CTD would clash electrostatically if it were not for the presence of a nucleotide neutralizing these like‐charges. In the absence of nucleotide, these clashes would have a destabilizing effect on the interface, causing the NTD to be released and swivel into an open conformation.

In order to design a mimic of the PPIs at the subunit interface, we aimed to rationally design a peptide that resembles the αF helix and neighbouring residues (Figure 2a). We envisaged that by anchoring the small molecule in the nucleotide binding site, the peptide part of the chimera would interact with the subunit–subunit interface, possibly displacing the native segment and in the process disrupting hexamer formation. The peptide sequence highlighted consists of the β9 sheet followed by a loop into the αF helix followed by a further loop into the β10 sheet (Figure 2b). It was unclear what length of peptide would be required to interact with the native peptide region, and therefore, three peptides with varying lengths were chosen for investigation: αF‐loop **1**, αF‐β10 **2**, and β9‐αF‐β10 **3** (Table 1). Furthermore, as Arg240 is in close proximity to the nucleotide binding site, this seemed to be the ideal site for conjugation to the imidazo[1,2‐*a*]pyrazine partner. By substituting this arginine for other amino acids capable of conjugating to other moieties, a range of bivalent inhibitors could be synthesized. We therefore prepared variants of these three peptides with Arg240 substituted by Cys (**4**, **5**, **6**), *S*‐allyl cysteine (SAC) (**7**, **8**, **9**), or azidolysine (AzLys) (**10**, **11**, **12**).

**TABLE 1:** Table indicating the amino acid sequence for each of the target peptides

Peptide	Sequence	WT	R240C	R240SAC	R240AzLys
αF‐loop	SADCLKSCLRMRPD	**1**	**4**	**7**	**10**
αF‐β10	SADCLKSCLRMRPDRIIL	**2**	**5**	**8**	**11**
β9‐αF‐β10	YTQLFFGGNITSADCLKSCLRMRPDRIIL	**3**	**6**	**9**	**12**

*Note*: Letters in red indicate an α‐helix; blue indicates a β‐sheet; black indicates turns and loops; R240 is highlighted in yellow.

Abbreviations: AzLys, azidolysine; SAC, *S*‐allyl cysteine.

**FIGURE 3:**
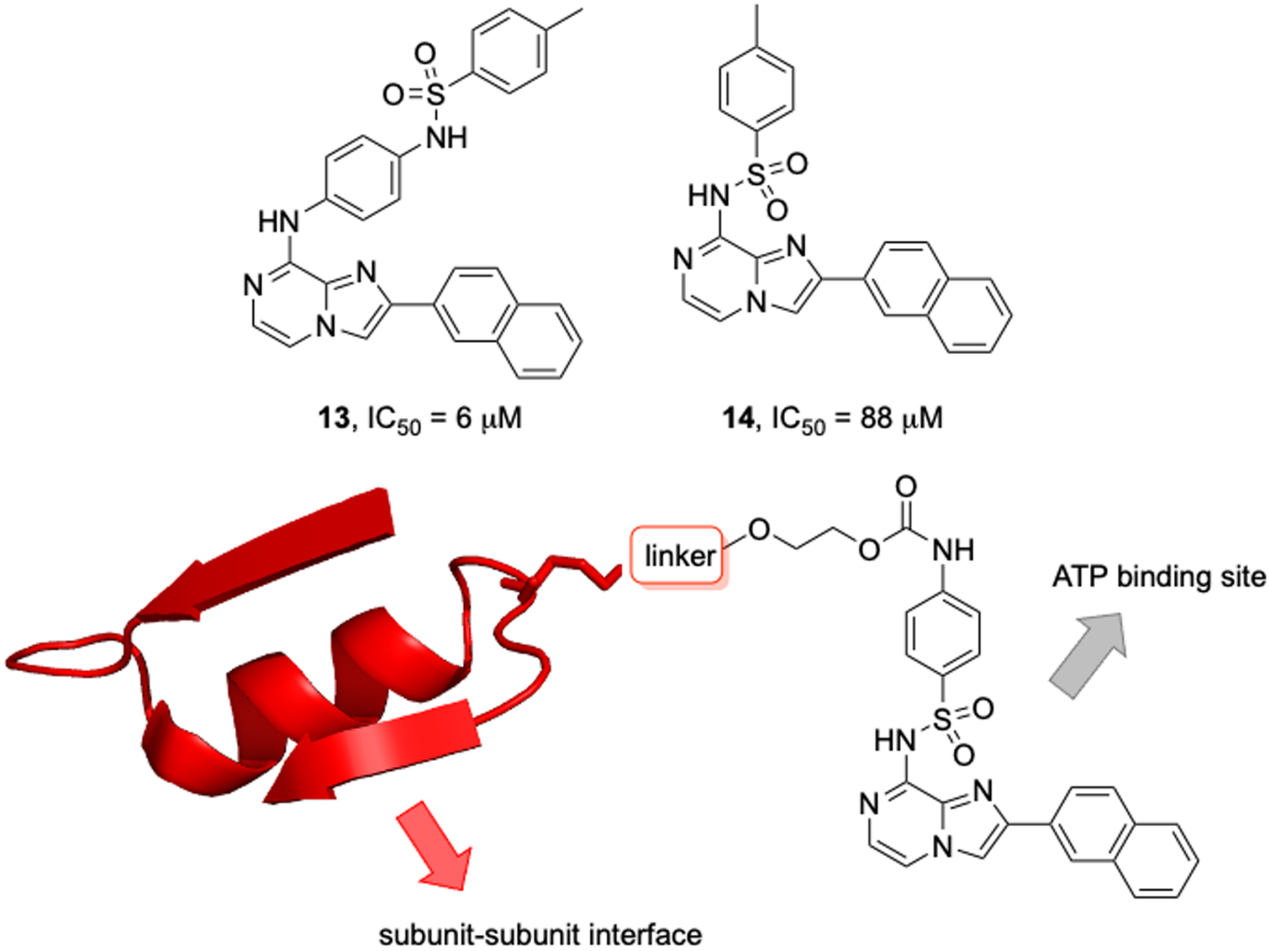
Structure of previously developed inhibitors and carbamate PEG linker design

We have previously reported a number of small molecule inhibitors of HP0525, based on the imidazo[1,2‐*a*]pyrazine scaffold (Figure 3), and carried out molecular docking studies on selected structures to elucidate their interactions with the active site.^30^ Docking studies of one of the best inhibitors, **13**, suggested that this series of inhibitors are deeply buried within the enzyme active site. Within the ATP‐binding site, the naphthalene ring occupies the purine binding region towards the solvent side (outside) of the enzyme, with the core imidazo[1,2‐*a*]pyrazine moiety occupying the ribose binding region. A sulfonamide group is also a common feature of this series of inhibitors, and in **13**, docking indicated that this sulfonamide moiety might occupy the phosphate binding region, pointing towards the adjacent subunit and the center of the hexameric chamber.^30^ This suggested that this region of these inhibitors might be an appropriate point for conjugation to the peptide recognition partner. Furthermore, modifications to this region of the inhibitors by attachment of a linker presented an opportunity to further explore the structure–activity relationships. We elected to modify the toluoyl moiety by adding a carbamate group and then linking the inhibitor to the peptide via an intermediate PEG chain. We reasoned that this would be more stable than an ester in vivo and would provide more hydrogen bonding possibilities in the phosphate binding/subunit–subunit interface region than an amide linkage. This in turn might potentially increase the potency and selectivity of this series of inhibitors. Our previous docking studies of another inhibitor in this series, **14**, had suggested that in this case, the sulfonamide points away from the phosphate region and out into the extra pocket (Figure S2). This leads to a difference in binding affinity and accounts for the drop in potency compared with **13**. However, the synthetic route to **14** was more direct and higher yielding than the route to **13** and easier to adapt for the preparation of carbamate‐linked bioconjugates. Moreover, the addition of the carbamate‐PEG linker was expected to afford bioconjugates of a similar length and spacing. We therefore elected to use **14** as the base imidazo[1,2‐*a*]pyrazine unit for initial investigation into bivalent HP0525 inhibitors.

Three of the most common approaches for peptide conjugation to the PEG chain were considered. Cross metathesis^46^ with the R240SAC peptides **7**, **8**, **9** incorporating *S*‐allyl cysteine could be carried out with **15.** Click chemistry^47^ requires azido lysine‐substituted peptidesR240AzLys **10**, **11**, **12** and could be achieved using alkyne **16**. Finally, maleimide coupling^48^ could be carried out between the cysteine‐substituted peptides **4**, **5**, **6** (R240C) and the maleimide **17**. The length of the PEG chain is also an important factor with the design of the bivalent inhibitor reagents. Preliminary docking studies suggested that a single PEG unit should be of sufficient length to extend from the carbamate linked to the toluoyl moiety, to the peptide sequence that will make interactions with the adjacent subunit.

Thus, our bivalent inhibitor reagents were designed to consist of a small molecule inhibitor, based on imidazo[1,2‐*a*]pyrazine, that will bind to the nucleotide binding site, linked (via a PEG chain) to a peptide, based on the αF helix of HP0525, that could then substitute the native αF helix and disrupt the opening and closing mechanism of the hexameric portal (Figure 1b).

### 3.2 Synthesis

There were a number of possible approaches to the synthesis of these bivalent inhibitors. We adopted the strategy of first synthesizing the imidazo[1,2‐*a*]pyrazine inhibitor, attached via a carbamate linker to a PEG chain primed with the conjugation moiety. These PEGylated‐imidazo[1,2‐*a*]pyrazines would then be regioselectively attached to a specific residue on the target peptides.

#### 3.2.1 Small molecule linker

**SCHEME 1:**
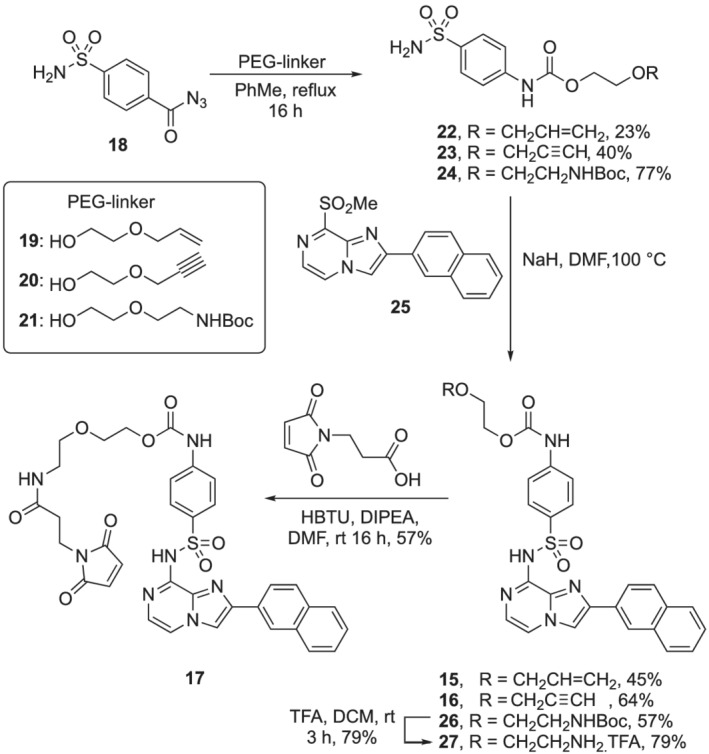
Synthetic route to PEGylated‐imidazo[1,2‐*a*]pyrazines **15**, **16**, and **17**

The synthesis of the allyl and alkyne linked PEGylated imidazo[1,2‐*a*]pyrazines (**15**, **16**) is shown in Scheme 1. The PEG‐linker was attached using a Curtius rearrangement of **18** (synthesized from the commercially available acid precursor)^49^ with PEG‐linkers **19**, **20**, **21** to give **22**, **23**, or **24**, respectively. The imidazo[1,2‐*a*]pyrazine fragment (**25**)^30^ was then incorporated using nucleophilic aromatic substitution. The Buchwald–Hartwig coupling used previously in the synthesis of **14**, to couple the sulfonamide portion to the chloro‐imidazo[1,2‐*a*]pyrazine fragment,^30^ was not suitable due to the presence of the alkene and alkyne.

PEGylated‐maleimide **17** was synthesized in an analogous manner to the alkyne and alkene linkers. Direct attachment of the maleimide to the PEG chain with an ethyl linker was unsuccessful, so an elongated carbamate structure was synthesized. The Buchwald–Hartwig coupling was low yielding due to the poor solubility of **24** (7% yield). Again, switching to nucleophilic displacement conditions (NaH and DMF) gave the desired product (**26**) in 57% yield. Removal of the Boc group afforded the free amine **27**, which was isolated as the TFA salt as decomposition was observed when the salt was basified. Finally, coupling of **27** to *N*‐maleoyl‐*β*‐alanine gave target maleimide **17**.

#### 3.2.2 Peptide synthesis

Initially, it was envisaged that chemoselective bioconjugation of the allyl (**15**) and maleimide (**17**) linked PEGylated imidazo[1,2‐*a*]pyrazines to appropriately substituted peptides would be carried out in solution. Peptides were therefore synthesized (Table 1) using Fmoc SPPS (Fmoc‐Asp (OtBu)‐NovaSyn®TGT and Fmoc‐Leu‐NovaSyn®TGT resins), incorporating either cysteine (**4**, **5**, **6**) or *S*‐allyl cysteine (SAC) (**7**, **8**, **9**) in the R240 position. Peptides **1**, **2**, and **3**, corresponding to the native sequence, were also synthesized in order to establish whether they adopted the predicted secondary structures. In the case of R240C peptides a non‐acid labile protecting group (acetamidomethyl ‐ Acm)^50^, ^51^ was used on the other cysteine residues so that the peptide produced has only one thiol free for maleimide conjugation.

#### 3.2.3 Bivalent inhibitor synthesis

**FIGURE 4:**
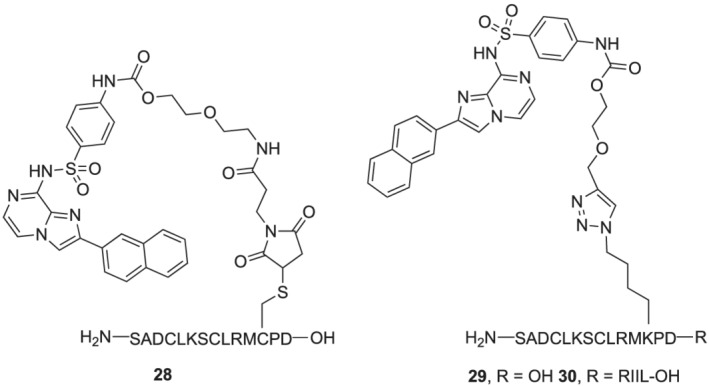
Structure of peptide conjugates synthesized

Before attempting to conjugate malemide **17** to the target peptides, reaction conditions were optimized using a model system. Reaction of Fmoc‐Cys‐OH with hexaethyleneglycol malemide in DMF gave the desired conjugated product in 81% yield. When these conditions were applied to the conjugation of **17** with peptides **4**, **5**, and **6**, MALDI analysis indicated that the desired conjugates had formed (see supporting information). However, not enough material was isolated after purification to continue with the Acm protecting group removal, and a large amount of unreacted **17** was recovered. Cross metathesis conditions were developed using *S*‐allyl cysteine and 2‐(allyloxy)ethanol (Grubbs' II catalyst^52^ [6 mol%] in ^*t*^BuOH/H_2_O at 32°C for 3 h, 100% conversion). However, when these conditions were applied to **15** and peptides **7** and **8**, no desired product (or starting peptides) was isolated. In order to overcome these difficulties, it was decided to conjugate the PEGylated imidazo[1.2‐*a*]pyrazines to the peptides on resin. The full length peptides were synthesized as before, but without the removal of the final Fmoc protecting group or global TFA deprotection/resin cleavage. In contrast to the solution phase synthesis, a trityl protecting group was used on the other cysteine residues, in place of the Acm group so that global deprotection would occur in the resin cleavage step. For the malemide coupling, the ^t^Bu protecting group on R240C was selectively removed using PBu_3_ in EtOH/CH_2_Cl_2_/H_2_O (4:6:1) for 3 h at RT.^53^ Conjugation of the free cysteine to **17** was then achieved by agitating in DMF at room temperature for 16 h, followed by standard Fmoc removal and deprotection/resin cleavage. This procedure was successful for the (αF‐loop peptide, giving **28** (Figure 4), but unfortunately no product was isolated when the longer peptides (αF‐β10 and β9‐αF‐β10) were used. Despite trialling a range of conditions, the on‐resin cross metathesis of R240SAC peptides and **15** was unsuccessful.

Greater success was seen with the alkyne tethered imidazo[1.2‐α]pyrazine **16**. The three desired peptides (αF‐loop **10**, αF‐β10 **11**, and β9‐αF‐β10 **12**) were synthesized on resin with azido lysine at the R240 position. The click reaction between R240AzLys peptides and **16** was carried out using excess CuI (20 eq) and sodium ascorbate (40 eq) in DMF/H_2_O/^*t*^BuOH (8:2:1) at room temperature for 16 h. After Fmoc removal and resin cleavage, both the conjugated αF‐loop (**29**) and αF‐β10 (**30**) peptides were isolated (Figure 4). However, no product could be isolated from the click reaction between resin‐bound β9‐αF‐β10 **12** and **16**.

### 3.3 Biological results

**FIGURE 5:**
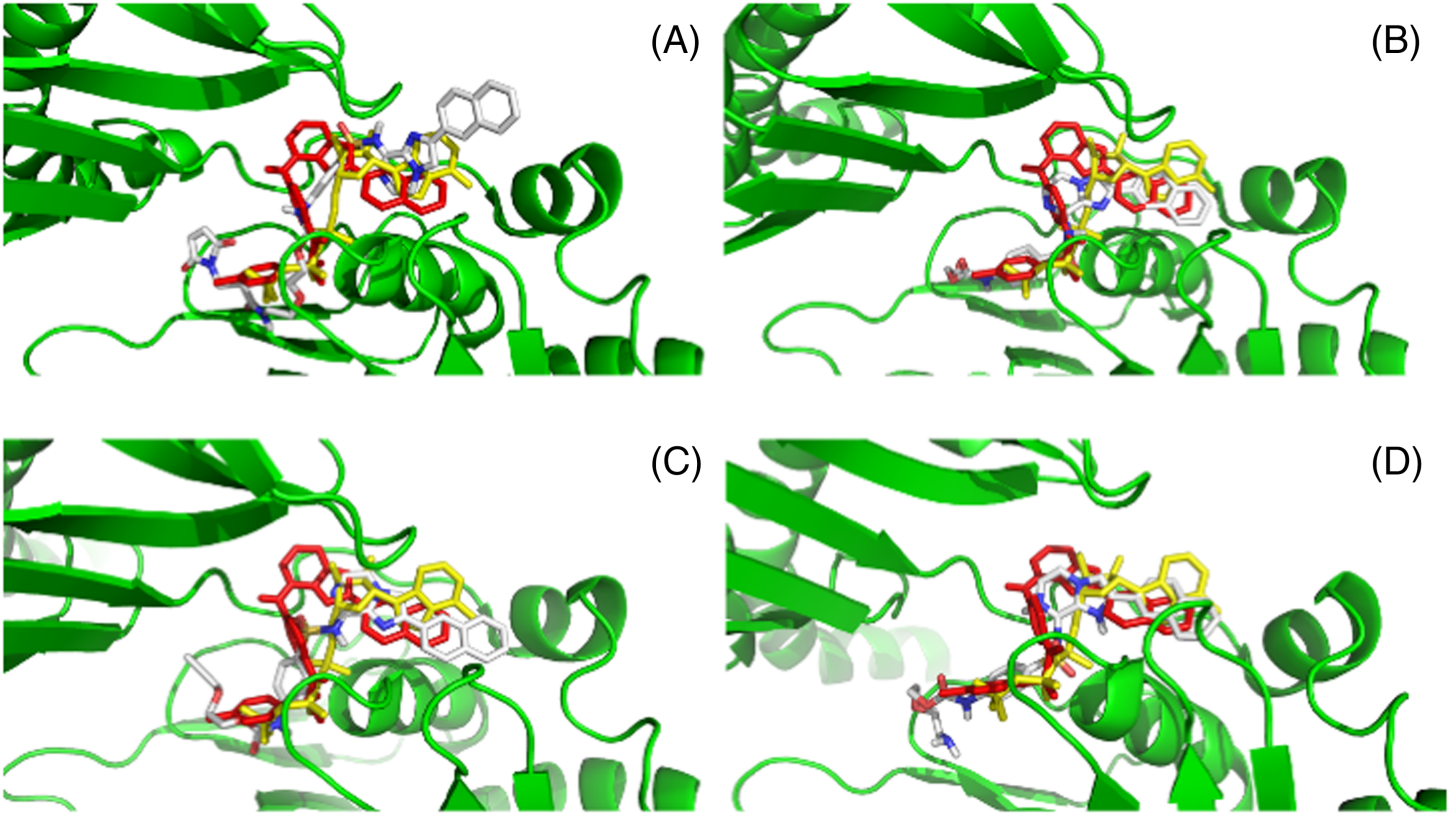
In silico images of **13** (red) and ATPγS (yellow) in ATPγS‐HP0525 with (A) **17**, (B) **15**, (C) **16**, (D) **27**. PDB: 1NLY. Image generated using PyMOL^45^

Initially, the activity of the PEGylated‐imidazo[1,2‐*a*]pyrazines were tested for inhibition of HP0525 using an in vitro ATPase colorimetric assay.^30^ The IC_50_ data are shown in Table 2. PEGylated‐imidazo[1,2‐*a*]pyrazines **15**, **16**, and **17** show improved inhibition of HP0525 compared with the parent compound **14**. In order to establish whether the extra H‐bonding possibilities provided by the carbamate moiety (and in the case of **17**, the amide and maleimide groups) had led to greater interactions with the active site, we carried out further docking studies (Figure 5). Indeed, when studying the in silico lowest energy binding modes within the nucleotide binding site, they appear to adopt a conformation similar to that of **13** rather than **14**. When looking at **17** (Figure 5A), the molecule shifts so that the carbamate mimics the same sulfonamide interactions as in **13**; the sulfonamide is now in the ribose region, and the imidazo[1,2‐*a*]pyrazine is located in the purine region. This means that the naphthalene moiety extends further into the enzyme active site, possibly picking up the further π‐stacking interactions; **15** and **16** have similar IC_50_ values, and this can be attributed to similar conformations observed within the nucleotide binding site (Figure 5B,C). It is interesting that **27** shows poor inhibition because in silico studies indicate similar conformations as **15** and **16** (Figure 5D), and therefore, a similar activity might have been expected.

**TABLE 2:** IC_50_ data for the PEGylated imidazo[1,2‐*a*]pyrazines synthesized

Compound	PEG chain (X)	IC_50_ (μM)
**14**	Carbamate replaced by CH_3_	88
**15**	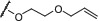	46
**16**	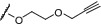	61
**27**	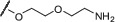	137
**17**	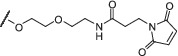	18

#### 3.3.1 Bivalent inhibitor reagents

The three peptide‐imidazo[1,2‐*a*]pyrazine conjugates (**28**, **29**, **30**) were tested at concentrations of 5, 50, and 500 μM, and the % absorbance at 620 nm analyzed to give an indication of their inhibitory effect (Table 3). The results clearly indicate that the peptide conjugates only inhibit very weakly at a high concentration.

**TABLE 3:** Percentage absorbance values for each of the peptide conjugates at 500, 50, and 5 μM

		% absorbance (620 nm)		
Entry	Peptide conjugate	500 μM	50 μM	5 μM
1	**28**	93	100	100
2	**29**	91	100	100
3	**30**	90	100	100

#### 3.3.2 Wild‐type peptides

**FIGURE 6:**
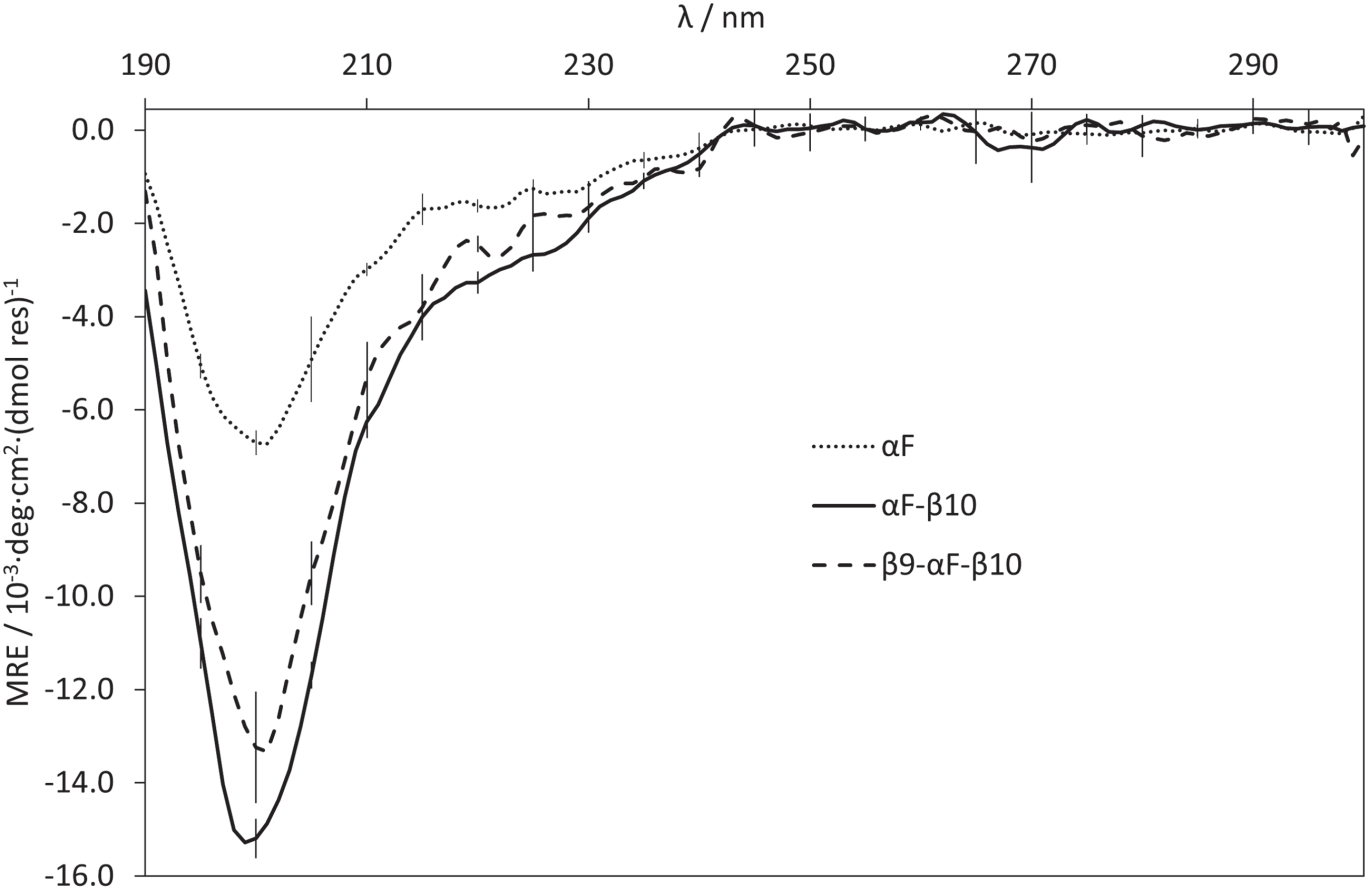
Smoothed CD spectra for the three different peptides, indicating the level of α‐helicity: αF‐loop **1**: 6%–7%, αF‐β10 **2**: 9%–10%, β9‐αF‐β10 **3**: 8%–9%. Spectra were processed with Dichroweb

The stability of the peptide fold might be important for both activity and reactivity. In silico stability testing on the three WT peptides using molecular dynamics showed only that the β9‐αF‐β10 peptide **3** was stable to the simulations (Figure S3); the αF‐loop peptide **1** unfolded immediately, and the αF‐β10 peptide **2** unfolded, but at a slower rate. Circular dichroism (CD) spectroscopy, however, indicated that none of the WT peptides synthesized form α‐helices (Figure 6).

## 4 CONCLUSION

Improving the selectivity profiles of ATPase or kinase inhibitors that bind to the ATP binding site is crucial to avoiding toxicity due to off‐target effects in vivo. A bivalent inhibitor approach, whereby the tight binding of ATP mimics is combined with targeting elements that bind outside the ATP binding site, has considerable potential. Designing a peptide or a small molecule that can mimic or disrupt PPIs is challenging, and the parameters that will lead to a successful inhibitor are not well‐understood.^54^

In this work, we have succeeded in improving the activity of a lead 8‐amino imidazo[1,2‐*a*]pyrazine inhibitor of the VirB11 ATPase HP0525 by attachment of a PEG moiety, primed for bioconjugation. We have established effective methodology for the bioconjugation of the 8‐amino imidazo[1,2‐*a*]pyrazine‐PEG to short peptides, via either cysteine‐maleimide reaction or alkyne/azide click chemistry. A possible reason for the conjugation failing for the longer length peptides could be the accessibility of the relevant amino acid. Where the conjugation was carried out on the solid phase, the R240 amino acid is close to the *C*‐terminus and therefore close to the resin. If the longer length peptides are extensively folded on‐resin, the thiol, allyl, or azide moieties might be considerably buried from their conjugation partner. The use of a lower loaded resin,^55^ or of protecting groups such as Hmb or pseudoproline moieties designed to disrupt folding on resin of “difficult sequences,^56^ could be beneficial, as could using alkyne/azide click chemistry for bioconjugation in solution.

However, conjugation to peptide fragments designed to mimic or disrupt the interactions between the subunits in the VirB11 ATPase HP0525 hexameric complex did not result in effective enzyme inhibitors. This was particularly disappointing, as functionalization of the 8‐amino imidazo[1,2‐*a*]pyrazine fragment with the PEG‐carbamate linkers actually improved their inhibition relative to the parent compound **14**. One possible explanation for this is that the linker between the 8‐amino imidazo[1,2‐*a*]pyrazine and the peptide fragment has the wrong length and/or structure to correctly orient the peptide fragment for binding to the next subunit. Whereas further detailed docking studies might help to identify improved linkers, a more powerful approach would undoubtably be to use a peptide aptamer “Trojan Horse”^57^ or an enzyme‐templated fragment elaboration strategy,^58^, ^59^ in which the enzyme itself selects the correct combination of linker and peptide fragment for effective bivalent binding from a small library. A second explanation is that the β9‐αF‐β10 segment of HP0525, and smaller fragments, do not fold in such a way as to form effective mimics of the HP0525 subunit interface, or that this segment cannot compete with the complete protein during assembly of the hexamer. This is partly supported by our CD and MD studies. Approaches such as helix stabilization by stapling^60^ might improve the binding, although introduction of a constraint to preorganize peptides to a particular secondary structure does not always increase the binding potency to the target.^61^ Finally, peptide sequences with *N*‐terminal capping and *C*‐terminal amide modifications should be synthesized, in order to avoid introducing additional charges to the sequence. All of these additional peptide modifications would then need to be tested separately for their ability to fold in solution, access the subunit–subunit interface, and to inhibit hexamer assembly. However, the initial studies presented in this paper will enable both an enzyme‐templated fragment elaboration strategy, and the pathway of the HP0525 hexameric complex assembly, to be further studied.

## Supporting information


**Data S1.** Supporting Information
