# Supplementary material for: Design, synthesis, and evaluation of peptide‐imidazo[1,2‐a]pyrazine bioconjugates as potential bivalent inhibitors of the VirB11 ATPase HP0525
Source: J Pept Sci. 2021 Jun 17;27(10):e3353. doi: 10.1002/psc.3353 (PMC12086749; doi:10.1002/psc.3353)
Supplement: Supplementary file 1 — Data S1. Supporting Information [file PSC-27-0-s001.pdf]

Design, synthesis and evaluation of peptide-imidazo[1,2-*a*]pyrazine bioconjugates as potential bivalent inhibitors of the VirB11 ATPase HP0525

James R. Sayer, Karin Walldén, Hans Koss, Helen Allan, Tina Daviter, Paul J. Gane, Gabriel Waksman, Alethea B. Tabor.*

Supporting Information

Contents

1. General Methods 3

2. Synthetic Procedures and Characterisation 4

4-Sulfamoylbenzoyl azide (**18**) 4

2-(Allyloxy)ethyl 4-sulfamoylphenylcarbamate (**22**) 4

2-(Prop-2-ynyloxy)ethyl 4-sulfamoylphenylcarbamate (**23**) 5

2-(2-(tert-butyl carbamate)ethoxy)ethyl 4-sulfamoylphenylcarbamate (**24**) 6

2-(Allyloxy)ethyl-4-(N-(2-(naphthalen-2-yl)imidazo[1,2-a]pyrazin-8-yl)sulfamoyl)phenylcarbamate (**15**) 7

2-(Prop-2-ynyloxy)ethyl-4-(N-(2-(naphthalen-2-yl)imidazo[1,2-a]pyrazin-8-yl)sulfamoyl)phenylcarbamate (**16**) 8

2-(tert-butylcarbamate-ethoxy)ethyl-4-(N-(2-(naphthalen-2-yl)imidazo[1,2-a]pyrazin-8-yl)sulfamoyl)phenylcarbamate (**26**) 9

2-(2-Aminoethoxy)ethyl-4-(N-(2-(naphthalen-2-yl)imidazo[1,2-a]pyrazin-8-yl)sulfamoyl)phenylcarbamate (**27**) 10

2-(2-(3-(Maleimido)propanamido)ethoxy)ethyl-4-(N-(2-(naphthalen-2-yl)imidazo[1,2-a]pyrazin-8-yl)sulfamoyl)phenylcarbamate (**17**) 11

3. HPLC Methods 13

Preparative HPLC 13

Analytical HPLC 14

4. Synthesis of Non-conjugated Peptides 15

αF-loop WT (**1**) 15

αF-loop R240C (**4**) 16

αF-loop R240SAC (**7**) 17

αF-β10 WT (**2**) 18

αF-β10 R240C (**5**) 19

αF-β10 R240SAC (**8**) 20

B9-αF-β10 WT (**3**) 21

B9-αF-β10 R240C (**6**) 22

B9-αF-β10 R240SAC (**9**) 23

5. Solution-Based Conjugation 24

αF-loop (**1** and **17**) 24

αF-β10 (**2** and **17**) 25

B9-αF-β10 (**3** and **17**) 26

6. Resin-Bound Conjugation 26

αF-loop (**28**) 26

αF-loop (**29**) 28

αF-β10 (**30**) 29

7. Figure S1 30

8. Figure S2 31

9. Figure S3 32

1. General Methods

Solvents and reagents were obtained from commercial sources and were used as received unless otherwise stated. Dry solvents were dried over anhydrous columns. Moisture levels were usually <15 ppm by Karl Fischer titration. Brine refers to a saturated solution of sodium chloride. Anhydrous magnesium sulfate ($MgSO_4$) or sodium sulfate (Na_2SO_4) were used as drying agents after reaction workup, as indicated. Pet. ether refers to the fraction of light petroleum ether boiling in the range 40-60 ºC.

Thin layer chromatography (TLC) was carried out using Fluka aluminium backed sheets coated with $60F_{254}$ silica gel. Visualisation of the silica plates was achieved using a UV lamp (λ_{max} = 245 nm) and/or potassium permanganate ($KMnO_4$ in 1M NaOH with 5% K_2CO_3). Flash chromatography was carried out using either Geduran (Merck) or ZEOprep (Apollo) Si60 40-63 µm silica gel.

Melting points (Mpt) were recorded on a Gallenkamp Melting Point Apparatus and are uncorrected. Infra-Red (IR) spectroscopy was carried out using a PerkinElmer Spectrum 100 FT-IR Spectrometer using thin films. Absorption maxima (ν_{max}) are reported in wavenumbers (cm^{-1}). Proton nuclear magnetic resonance (^1H NMR) were recorded using Bruker AV400 (400 MHz), AV500 (500 MHz) and AV600 (600 MHz) spectrometers as indicated. Carbon nuclear magnetic resonance (^{13}C NMR) were recorded using Bruker AV400 (100 MHz), AV500 (125 MHz) and AV600 (150 MHz) spectrometers as indicated. Spectra were obtained using CD_3OD, CD_3CN and $(CD_3)_2SO$ as solvents and chemical shifts are quoted on the δ scale in units of ppm using TMS as an internal standard. Coupling constants (*J*) are reported in Hz with the following splitting abbreviations: s (singlet), d (doublet), t (triplet), q (quartet), dd (doublet of doublets), b (broad), dt (doublet of triplets), m (multiplet), ap. (apparent). Compounds were characterised with the aid of 2D spectra (COSY, HSQC, HMBC and NOESY).

High Performance Liquid Chromatography (HPLC) was performed using a Varian ProStar instrument; DiscoveryBIO wide pore C18-10 (25 cm x 4.6 mm, 10 µm) for reverse phase analytical HPLC; and a DiscoveryBIO wide pore C18 (25 cm x 21.2 mm, 10 µm) column for reverse phase preparative HPLC. Each solvent used contained 0.1% TFA buffer.

Liquid Chromatography Mass Spectrometry (LCMS) was carried out using SQD-Waters Acquity UPLC/SQD with C18 (50 mm x 2.1 mm, 1.7 µm) column. A total run time of 5 minutes and flow rate of 0.6 mL/min was used with gradient elution: 95% H_2O/ 5% MeCN (0

min), 5% H_2O/ 95% MeCN (3 min), 95% H_2O/ 5% MeCN (4.5 min). Each solvent contained 0.1% formic acid buffer. LRMS refers to low resolution mass spectrometry and HRMS refers to high resolution mass spectrometry. Electron Impact/ Chemical Ionisation (EI/CI) MS was carried out using MAT900XP (Thermo Finnigan) instrument and electrospray ionization (ES) accurate mass was determined using Waters LCT Premier XE instrumentation.

2. Synthetic Procedures and Characterisation

4-Sulfamoylbenzoyl azide (**18**)

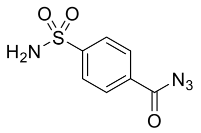

4-Sulfamoylbenzoic acid (1.00 g, 4.97 mmol), PPh_3 (2.61 g, 9.94 mmol) and NaN_3 (0.388 g, 5.96 mmol) were suspended in anhydrous acetone (10 mL). To this milky white suspension was added trichloroacetonitrile (0.997 mL, 9.94 mmol) dropwise and the reaction was left to stir at RT for 18 h. The solvent was removed *in vacuo* (no heat) and the resulting dark yellow slurry was diluted with CH_2Cl_2 (100 mL) and washed with H_2O (60 mL) and brine (50 mL), dried ($MgSO_4$), filtered and concentrated *in vacuo*. Flash chromatography (applied in pet. ether; eluted 3:1 to 2:1 to 1:1 pet. ether/EtOAc) afforded the title compound as a white solid (907 mg, 4.01 mmol, 80.7%). Mpt: 118 °C; R_f = 0.23 (1:1 pet. ether/EtOAc); IR (ν_{max}/cm^{-1}, thin film): 3362 (N-H stretch), 3258 (aromatic C-H stretch), 2137 (N=N=N stretch), 1687 (C=O stretch), 1339 (S=O asymmetric stretch), 1238, 1155 (S=O symmetrical stretch); ^{1}H NMR (600 MHz, CD_3OD): δ_H = 8.01-8.02 (m, 2H, **4-H**), 8.17-8.18 (m, 2H, **3-H**); ^{13}C NMR (150 MHz, CD_3OD): δ_C = 127.6 (**C-4**), 131.0 (**C-3**), 134.9 (**C-2**), 150.2 (**C-5**), 172.8 (**C-1**); Anal. Calcd. for $C_7H_6N_4O_3S$: C, 37.17; H, 2.67; N, 24.77. Found C, 37.27; H, 2.49; N, 24.40%.

2-(Allyloxy)ethyl 4-sulfamoylphenylcarbamate (**22**)

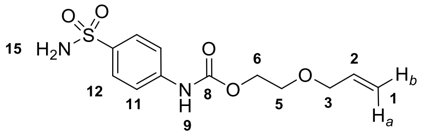

To a suspension of 4-sulfamoylbenzoic acid (2.01 g, 10.0 mmol) in anhydrous toluene (15 mL) was added Et_3N (1.63 mL, 11.7 mmol). Diphenyphopshoryl azide (2.52 mL, 11.7 mmol) in

anhydrous toluene (10 mL) was added dropwise to the reaction mixture and stirred at RT for 30 min followed by heating at 90 °C for 30 min. 2-(allyloxy)ethanol (1.28 mL, 12.0 mmol) in anhydrous DMF (6 mL) was then added and the reaction was stirred at 90 °C for 16 h. The reaction was cooled to RT and solvent removed *in vacuo*. The crude material was taken up in EtOAc (100 mL) and washed with $NaHCO_3$ (sat. aq. 50 mL), H_2O (3 x 40 mL) and brine (40 mL); dried ($MgSO_4$), filtered and concentrated *in vacuo* to give an orange/brown sticky solid. Flash chromatography (applied in pet. ether; eluted 2:1 to 1:1 pet. ether/EtOAc) afforded the title compound as a white solid (689 mg, 2.30 mmol, 23%). Mpt: 128 °C; R_f = 0.41 (2:1 EtOAc/pet. ether); IR (ν_{max}/cm^{-1}, thin film): 3356, 3298, 3193, 3110, 2908 (C-H and N-H stretches), 1734 (C=O stretch), 1596 (C=C stretch), 1529 (N-H bend), 1336 (S=O asymmetric stretch), 1314, 1216, 1151 (S=O symmetric stretch), 1099, 1055 (C-O stretches); ^{1}H NMR (600 MHz, $(CD_3)_2SO$): δ_H = 3.63-3.64 (m, 2H, **5-H**), 3.99 (dt, *J* = 5.3, 1.6 Hz, 2H, **3-H**), 4.24-4.25 (m, 2H, **6-H**), 5.16 (dd, *J* = 10.4, 1.6 Hz, 1H, **1-H*b***), 5.27 (dd, *J* = 17.3, 1.6 Hz, 1H, **1-H*a***), 5.85-5.91 (m, 1H, **2-H**), 7.23 (s, 2H, **15-H**), 7.62 (d, *J* = 8.8 Hz, 2H, **11-H**), 7.72-7.72 (m, 2H, **12-H**), 10.17 (s, 1H, **9-H**); ^{13}C NMR (150 MHz, $(CD_3)_2SO$): δ_C = 63.9 (**C-6**), 67.7 (**C-5**), 71.0 (**C-3**), 116.7 (**C-1**), 117.7 (**C-11**), 126.8 (**C-12**), 135.0 (**C-2**), 137.6 (**C-13**), 142.3 (**C-10**), 153.4 (**C-8**); LRMS m/z (EI$^+$): 300 [M]$^-$, 243 [M-$OCH_2CH=CH_2$]; HRMS m/z (EI$^+$): Found 300.07810 [M]$^+$; $C_{12}H_{16}N_2O_5S$ requires 300.07744; Anal. Calcd. for $C_{12}H_{16}N_2O_5S$: C, 47.99; H, 5.37; N, 9.33. Found C, 48.24; H, 5.27; N, 9.57%.

2-(Prop-2-ynyloxy)ethyl 4-sulfamoylphenylcarbamate (23)

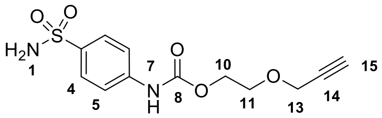

20 (557 mg, 5.57 mmol) was dissolved in anhydrous toluene (140 mL). **18** (1.51 g, 6.68 mmol) and molecular sieves (4Å, 10 sieves) were added and the reaction was stirred under reflux for 16 h. The solvent was removed and the reaction purified *via* flash chromatography (applied in toluene; eluted 2:1 toluene/EtOAc) to afford the title compound as a white solid (663 mg, 2.22 mmol, 40%). Mpt: 100-102 °C; R_f = 0.17 (2:1 toluene/EtOAc); IR (ν_{max}/cm^{-1}, thin film): 3350 (≡C-H stretch), 3273 (aromatic C-H stretch), 2116 (C≡C stretch), 1704 (C=O stretch), 1595, 1533 (N-H bends), 1314 (S=O asymmetric stretch), 1234 (COOR stretch), 1152 (S=O symmetric stretch), 1067 (C-O stretch); ^{1}H NMR (600 MHz, $(CD_3)_2SO$): δ_H = 3.48 (t, *J* = 2.3 Hz, 1H, **15-H**), 3.69-3.70 (m, 2H, **11-H**), 4.20 (d, *J* = 2.3 Hz, 2H, **13-H**), 4.24-4.26 (m, 2H, **10-**

H), 7.23 (s, 2H, **1-H**), 7.61 (d, J = 8.8 Hz, 2H, **5-H**), 7.71-7.73 (m, 2H, **4-H**), 10.18 (s, 1H, **7-H**); ^{13}C NMR (150 MHz, $(CD_3)_2SO$): δ_C = 57.5 (**C-13**), 63.6 (**C-10**), 67.3 (**C-11**), 77.5 (**C-15**), 80.1 (**C-14**), 117.6 (**C-5**), 126.8 (**C-4**), 137.6 (**C-3**), 142.2 (**C-6**), 153.3 (**C-8**); LRMS m/z (EI$^+$): 298 [M]$^+$, 243 [M-$OCH_2C{\equiv}CH$]$^+$, 216 [M-$CH_2CH_2OCH_2C{\equiv}CH$]$^+$, 183 [M-C(O)$OCH_2CH_2OCH_2C{\equiv}CH$]$^+$; HRMS m/z (EI$^+$): Found 298.06247 [M]$^+$; $C_{12}H_{14}N_2O_5S$ requires 298.06179; Anal. Calcd. for $C_{12}H_{14}N_2O_5S$: C, 48.31; H, 4.73; N, 9.39. Found C, 48.60; H, 4.66; N, 9.12%.

2-(2-(tert-butyl carbamate)ethoxy)ethyl 4-sulfamoylphenylcarbamate (**24**)

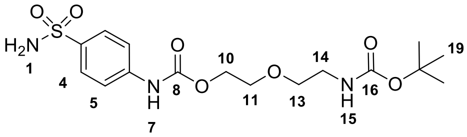

21 (993 mg, 4.39 mmol) was dissolved in anhydrous toluene (92 mL). **18** (751 mg, 3.66 mmol) was added and the reaction was stirred under reflux for 16 h. The reaction was cooled to RT, solvent removed and purification *via* flash chromatography (pet. ether; 2:1 to 1:1 to 1:2 pet. ether/EtOAc) afforded the title compound as a white solid (1.13 g, 2.81 mmol, 77%). Mpt: 94-95 °C; R_f = 0.35 (1:3 pet. ether/EtOAc); IR (ν_{max}/cm^{-1}, thin film): 3368, 3332 (aromatic C-H stretch), 3201, 3101, 2976, 2919 (C-H, N-H stretches), 1703, 1676 (C=O stretches), 1522 (N-H bend), 1333 (S=O asymmetric stretch), 1243 (COOR stretch), 1156 (S=O symmetric stretch), 1073 (C-O stretch); ^{1}H NMR (600 MHz, CD_3OD): δ_H = 1.41 (s, 9H, **19-H**), 3.22 (t, J = 5.7 Hz, 2H, **14-H**), 3.53 (t, J = 5.7 Hz, 2H, **13-H**), 3.71 (t, J = 4.7 Hz, 2H, **11-H**), 4.29 (t, J = 4.7 Hz, 2H, **10-H**), 7.60 (d, J = 8.8 Hz, 2H, **5-H**), 7.81 (ap.d, J = 8.8 Hz, 2H, **4-H**); ^{13}C NMR (150 MHz, CD_3OD): δ_C = 28.8 (**C-19**), 41.3 (**C-14**), 65.4 (**C-10**), 70.2 (**C-11**), 71.0 (**C-13**), 80.2 (**C-18**), 119.0 (**C-5**), 128.3 (**C-4**), 138.7 (**C-3**), 144.1 (**C-6**), 155.4 (**C-8**), 158.2 (**C-16**); LRMS m/z (ES$^+$): 426 [M+Na]$^+$, 370 [M-^{t}Bu+Na]$^+$; HRMS m/z (ES$^+$): Found 426.1306 [M+Na]$^+$; $C_{16}H_{25}N_3O_7NaS$ requires 426.1311; Anal. Calcd. for $C_{16}H_{25}N_3O_7S$: C, 47.63; H, 6.25; N, 10.42. Found C, 47.78; H, 6.46; N, 10.20%

2-(Allyloxy)ethyl-4-(N-(2-(naphthalen-2-yl)imidazo[1,2-a]pyrazin-8-yl)sulfamoyl)phenyl-carbamate (**15**)

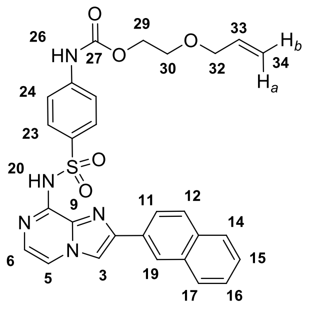

NaH was pre-activated by stirring NaH (60% in Mineral Oil; 70.6 mg, 1.77 mmol) in anhydrous hexanes (50 mL) for 20 min, removing the solvent using a syringe and drying the contents under high vacuum. DMF (5 mL) was added followed by **22** (529 mg, 1.77 mmol) in DMF (7 mL) and the mixture was stirred at RT for 20 min. **25** (285 mg, 0.882 mmol) in DMF (13 mL) was added and the resulting dark brown solution was heated at 100 °C under Ar for 16 h. The mixture was then diluted with EtOAc (100 mL) and washed with NH_4Cl (sat. aq. 50 mL) and H_2O (5 x 40 mL). The combined aqueous layers were then re-extracted with EtOAc (2 x 40 mL), followed by washing the combined organics with brine (40 mL) drying ($MgSO_4$), filtering and concentrating *in vacuo*. Flash chromatography (applied in CH_2Cl_2; eluted 10% to 20% to 33% EtOAc/CH_2Cl_2) afforded the title compound as a light yellow solid (214 mg, 0.395 mmol, 45%). Mpt: >200 °C; R_f = 0.25 (1:1 CH_2Cl_2/EtOAc); IR (ν_{max}/cm^{-1}, thin film): 3223 (aromatic C-H stretch), 1720 (C=O stretch), 1583 (N-H bend and C=C stretch), 1392 (S=O asymmetric stretch), 1112 (S=O symmetric stretch), 1065 (C-O stretch); ^{1}H NMR (600 MHz, $(CD_3)_2SO$): δ_H = 3.62 (dt, *J* = 4.6, 3.1 Hz, 2H, **30-H**), 3.97 (d, *J* = 6.7 Hz, 2H, **32-H**), 4.23 (ap.t, *J* = 4.4 Hz, 2H, **29-H**), 5.14 (dd, *J* = 10.5, 1.6 Hz, 1H, **34-H*b***), 5.25 (dd, *J* = 17.3, 1.6 Hz, 1H, **34-H*a***), 5.84-5.91 (m, 1H, **33-H**), 7.16 (d, *J* = 5.2 Hz, 1H, **6-H**), 7.50-7.55 (m, 2H, **15,16-H**), 7.65 (d, *J* = 8.7 Hz, 2H, **24-H**), 7.86 (d, *J* = 5.2 Hz, 1H, **5-H**), 7.92 (d, *J* = 8.5 Hz, 3H, **14,23-H**), 7.98 (d, *J* = 8.5 Hz, 1H, **12-H**), 8.03 (t, *J* = 8.9 Hz, 2H, **11,17-H**), 8.52 (s, 1H, **19-H**), 8.59 (s, 1H, **3-H**), 10.21 (s, 1H, **26-H**), 11.63 (bs, 1H, **7/20-H**); ^{13}C NMR (150 MHz, $(CD_3)_2SO$): δ_C = 63.9 (**C-29**), 67.7 (**C-30**), 71.0 (**C-32**), 110.9 (**C-5**), 115.3 (**C-3**), 116.7 (**C-34**), 116.8 (**C-6**), 117.6 (**C-24**), 123.8 (**C-11**), 124.2 (**C-19**), 126.3 (**C-15**), 126.6 (**C-16**), 127.4 (**C-23**), 127.7 (**C-14**), 128.3 (**C-17**), 128.4 (**C-12**), 130.0 (**C-10**), 132.8 (**C-13**), 133.2 (**C-18**), 135.0 (**C-33**), 135.6 (**C-9**) 135.9 (**C-22**), 142.9 (**C-25**), 144.4 (**C-8**), 145.2 (**C-2**), 153.3 (**C-27**),; LRMS m/z (ES$^+$):

544 [M+H]$^+$; HRMS m/z (ES$^+$): Found 544.1631 [M+H]$^+$; $C_{28}H_{26}N_5O_5S$ requires 544.1655; Anal. Calcd. for $C_{28}H_{25}N_5O_5S$: C, 61.87; H, 4.64; N, 12.88. Found C, 61.61; H, 4.32; N, 12.85%

2-(Prop-2-ynyloxy)ethyl-4-(N-(2-(naphthalen-2-yl)imidazo[1,2-a]pyrazin-8-yl)sulfamoyl)phenylcarbamate (16)

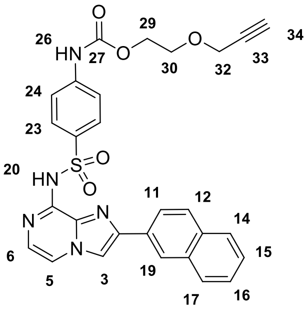

NaH was pre-activated by stirring NaH (60% in Mineral Oil; 88.7 mg, 2.22 mmol) in anhydrous hexanes (25 mL) for 20 min, removing the solvent using a syringe and drying the contents under high vacuum. DMF (6 mL) was added followed by **23** (660 mg, 2.22 mmol) in DMF (8 mL) and the mixture was stirred at RT for 20 min. **25** (358 mg, 1.11 mmol) in DMF (20 mL) was added and the resulting dark brown solution was heated at 100 °C under Ar for 18 h. The solvent was removed and the resulting residue was then taken up in EtOAc (100 mL) and washed with NH_4Cl (sat. aq. 50 mL) and H_2O (5 x 40 mL). The combined aqueous layers were then re-extracted with EtOAc (2 x 40 mL), followed by washing the combined organics with brine (40 mL), drying ($MgSO_4$), filtering and concentrating *in vacuo*. Flash chromatography (applied in pet. ether; eluted 1:1 to 1:2 to 1:4 to 1:9 pet. ether/EtOAc) afforded the title compound as a yellow/orange solid (380 mg, 0.703 mmol, 64%). Mpt: 184-186 °C; R_f = 0.32 (9:1 EtOAc/pet. ether); IR (ν_{max}/cm^{-1}, thin film): 3227 (aromatic C-H and ≡C-H stretch), 2112 (C≡C stretch), 1719 (C=O stretch), 1582, 1532 (N-H bends), 1391 (S=O asymmetric stretch), 1222 (COOR stretch), 1141 (S=O symmetric stretch), 1112 (C-O stretch); ^{1}H NMR (600 MHz, $(CD_3)_2SO$): δ_H = 3.46 (t, J = 2.4 Hz, 1H, **34-H**), 3.67-3.69 (m, 2H, **30-H**), 4.18 (d, J = 2.4 Hz, 2H, **32-H**), 4.23-4.25 (m, 2H, **29-H**), 7.16 (t, J = 5.5 Hz, 1H, **6-H**), 7.51-7.54 (m, 2H, **15,16-H**), 7.65 (d, J = 8.9 Hz, 2H **24-H**), 7.86 (d, J = 5.6 Hz, 1H, **5-H**), 7.92-7.93 (m, 3H, **14,23-H**), 7.98 (d, J = 8.6 Hz, 1H, **12-H**), 8.02-8.05 (m, 2H, **11,17-H**), 8.52 (s, 1H, **19-H**), 8.59 (s, 1H, **3-H**), 10.22 (s, 1H, **26-H**), 11.63 (bd, J = 4.7 Hz, **7/20-H**); ^{13}C NMR (150 MHz, $(CD_3)_2SO$): δ_C = 57.5 (**C-32**), 63.7 (**C-29**), 67.3 (**C-30**), 77.5 (**C-34**), 80.1 (**C-33**), 110.9 (**C-5**), 115.3 (**C-3**),

116.8 (**C-6**), 117.7 (**C-24**), 123.8 (**C-11**), 124.2 (**C-19**), 126.3 (**C-15**), 126.6 (**C-16**), 127.4 (**C-23**), 127.7 (**C-14**), 128.3 (**C-17**), 128.4 (**C-12**), 130.0 (**C-10**), 132.8 (**C-13**), 133.2 (**C-18**), 135.6 (**C-9**), 136.9 (**C-22**), 142.9 (**C-25**), 144.4 (**C-8**), 145.2 (**C-2**), 153.3 (**C-27**); LRMS m/z (ES$^+$): 542 [M+H]$^+$; HRMS m/z (ES$^+$): Found 542.1512 [M+H]$^+$; $C_{28}H_{24}N_5O_5S$ requires 542.1498; Anal. Calcd. for $C_{28}H_{23}N_5O_5S$: C, 62.10; H, 4.28; N, 12.93. Found C, 65.36; H, 3.66; N, 12.62%.

2-(tert-butylcarbamate-ethoxy)ethyl-4-(N-(2-(naphthalen-2-yl)imidazo[1,2-a]pyrazin-8-yl)-sulfamoyl)phenylcarbamate (26)

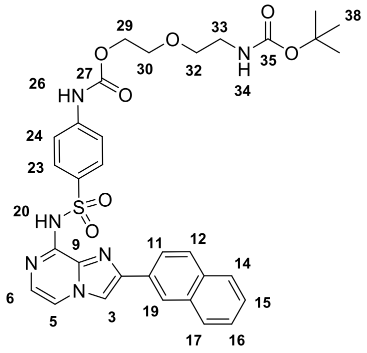

NaH was pre-activated by stirring NaH (60% in Mineral Oil; 71.0 mg, 1.77 mmol) in anhydrous hexanes (25 mL) for 20 min, removing the solvent using a syringe and drying the contents under high vacuum. DMF (5 mL) was added followed by **24** (715 mg, 1.77 mmol) in DMF (8 mL) and the mixture was stirred at RT for 20 min. **25** (287 mg, 0.887 mmol) in DMF (17 mL) was added and the resulting dark brown solution was heated at 100 °C under Ar for 16 h. The solvent was then removed and the residue was diluted with EtOAc (100 mL) and washed with NH_4Cl (aq. Sat 50 mL) and H_2O (3 x 40 mL). The combined aqueous layers were then re-extracted with EtOAc (2x), followed by washing the combined organics with brine, drying ($MgSO_4$), filtering and concentrating *in vacuo*. Flash chromatography (applied in pet. ether; eluted 1:1 to 1:2 to 1:3 to 1:4 to 1:5 pet. ether/EtOAc) afforded the title compound as a light yellow solid (328 mg, 0.507 mmol, 57%). Mpt: >200 °C; R_f = 0.21 (9:1 EtOAc/pet. ether); IR (ν_{max}/cm^{-1}, thin film): 3230 (C-H stretch), 2972 (N-H stretch), 1697 (C=O stretch), 1584, 1530 (N-H bend), 1393 (S=O asymmetric stretch), 1221, 1114 (S=O symmetric stretch). 1064 (C-O stretch); ^{1}H NMR (600 MHz, $(CD_3)_2SO$): δ_H = 1.34 (s, 9H, **38-H**), 3.06 (ap.q, J = 5.5 Hz, 2H, **33-H**), 3.47 (t, J = 5.5 Hz, 2H, **32-H**), 3.62 (t, J = 4.3 Hz, 2H, **30-H**), 4.20 (t, J = 4.3 Hz, 2H,

29-H), 6.80 (t, J = 5.5 Hz, 1H, **34-H**), 7.16 (bs, 1H, **6-H**), 7.50-7.55 (m, 2H, **15,16-H**), 7.65 (d, J = 8.8 Hz, 2H, **24-H**), 7.86 (bd, J = 4.6 Hz, 1H, **5-H**), 7.92 (d, J = 7.4 Hz, 3H, **14,23-H**), 7.98 (d, J = 8.6 Hz, 1H, **12-H**), 8.02-8.05 (m, 2H, **11,17-H**), 8.52 (s, 1H, **19-H**), 8.59 (s, 1H, **3-H**), 10.22 (s, 1H, **26-H**), 11.63 (bs, 1H, **7/20-H**); ^{13}C NMR (150 MHz, $(CD_3)_2SO$): δ_C = 28.3 (**C-38**), 40.0 (**C-33**), 63.9 (**C-29**), 68.2 (**C-30**), 69.1 (**C-32**), 77.7 (**C-37**), 110.9 (**C-5**), 115.3 (**C-**3), 116.8 (**C-6**), 117.6 (**C-24**), 123.8 (**C-11**), 124.2 (**C-19**), 126.3 (**C-15**), 126.6 (**C-16**), 127.4 (**C-23**), 127.7 (**C-14**), 128.3 (**C-12**), 128.4 (**C-17**), 130.0 (**C-10**), 132.8 (**C-13**), 133.2 (**C-18**), 135.6 (**C-9**), 135.8 (**C-22**), 142.9 (**C-25**), 145.2 (**C-2**), 153.3 (**C-27**), 155.6 (**C-35**) 145.5 (**C-8**); LRMS m/z (EI$^+$): 647 [M+H]$^+$, 591 [M-^{t}Bu]$^+$; (ES$^-$): 691 [M+Formic Acid]$^+$, 645 [M-H]$^-$; HRMS m/z (ES$^-$): Found 645.2140 [M-H]$^-$; $C_{32}H_{33}N_6O_7S$ requires 645.2131; Anal. Calcd. for $C_{32}H_{34}N_6O_7S$: C, 59.43; H, 5.30; N, 12.99. Found C, 58.35; H, 5.39; N, 12.34%.

2-(2-Aminoethoxy)ethyl-4-(N-(2-(naphthalen-2-yl)imidazo[1,2-a]pyrazin-8-yl)sulfamoyl)phenylcarbamate (27)

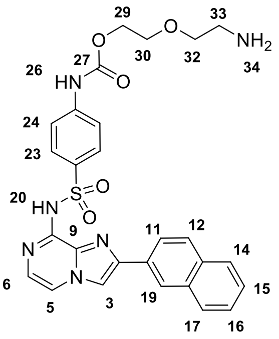

26 (318 mg, 0.492 mmol) was dissolved in anhydrous CH_2Cl_2 (15 mL) and cooled on ice. TFA (15 mL) was added and the reaction was stirred at RT for 3 h. Removal of the solvent, *via* trituration with toluene, followed by flash chromatography (applied in CH_2Cl_2; eluted 5% to 10% to 20% MeOH/CH_2Cl_2) afforded the title compound as an off white solid (390 mg, 0.389 mmol, 79%). Mpt: >200 °C; R_f = 0.26 (20% MeOH/CH_2Cl_2); IR (ν_{max}/cm^{-1}, thin film): 1726 (C=O stretch), 1675 (N-H bend), 1351 (S=O asymmetric stretch), 1200, 1120 (S=O symmetric stretch), 1071 (C-O stretch); ^{1}H NMR (600 MHz, $(CD_3)_2SO$): δ_H = 2.99 (t, J = 5.3 Hz, 2H, **33-H**), 3.62 (t, J = 5.3 Hz, 2H, **32-H**), 3.69 (t, J = 4.5 Hz, 2H, **30-H**), 4.24 (t, J = 4.5 Hz, 2H, **29-H**), 7.14 (d, J = 5.0 Hz, 1H, **6-H**), 7.49-7.54 (m, 2H, **15,16-H**), 7.58 (d, J = 8.5 Hz, 2H, **24-H**), 7.80 (d, J = 5.0 Hz, 1H, **5-H**), 7.89-7.92 (m, 3H, **14,23-H**), 7.97 (d, J = 8.6 Hz, 1H, **12-H**),

8.01 (d, *J* = 7.9 Hz, 1H, **17-H**), 8.05 (dd, *J* = 8.6, 1.4 Hz, 1H, **11-H**), 8.49 (s, 1H, **3-H**), 8.52 (s, 1H, **19-H**), 10.09 (bs, 1H, **20-H**); ^{13}C NMR (150 MHz, $(CD_3)_2SO$): δ_C = 38.6 (**C-33**), 63.6 (**C-29**), 66.7 (**C-32**), 68.5 (**C-30**), 110.7 (C-**5**) 116.3 (**C-3**), 117.4 (**C-24**), 118.3 (**C-6**), 123.9 (**C-11**), 124.0 (**C-19**) 126.1 (**C-15**), 126.5 (**C-16**) 127.7 (overlapping signals, **C-14,23**), 128.2 (**C-17**), 128.3 (**C-12**), 130.6 (**C-10**), 132.7 (**C-13**), 133.6 (**C-18**), 136.2 (**C-9**), 137.4 (**C-22**), 142.0 (**C-25**), 144.2 (**C-2**), 149.7 (**C-8**), 153.5 (**C-27**); LRMS m/z (ES^+): 547 $[M+H]^+$; HRMS m/z (ES^+): Found 547.1757 $[M+H]^+$; $C_{27}H_{27}N_6O_5S$ requires 547.1764; Anal. Calcd. for $C_{35}H_{30}F_{12}N_6O_{13}S$ (4x TFA): C, 41.92; H, 3.02; N, 8.38. Found C, 41.14; H, 2.98; N, 8.71%.

2-(2-(3-(Maleimido)propanamido)ethoxy)ethyl-4-(N-(2-(naphthalen-2-yl)imidazo[1,2-a]-pyrazin-8-yl)sulfamoyl)phenylcarbamate (**17**)

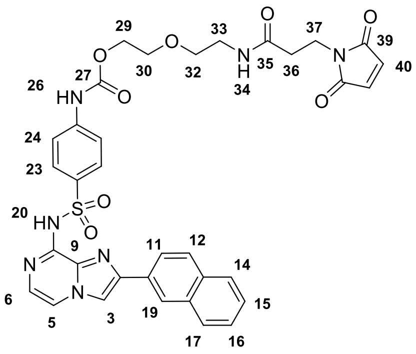

3-Maleimidopropanoic acid (62.7 mg, 0.371 mmol) and HBTU (211 mg, 0.556 mmol) were dissolved in anhydrous DMF (15 mL) and the mixture was purged with Ar. DIPEA (197 µL, 1.11 mmol) was added and a colourless to orange colour change was observed after stirring at RT for 20 min. **27** (367 mg, 0.556 mmol) in anhydrous DMF (5 mL) was then added and the reaction was stirred at RT for 16 h. Removal of the solvent *in vacuo* was followed by diluting with EtOAc (50 mL) and washing with H_2O (4 x 30 mL) and brine (30 mL), drying ($MgSO_4$), filtering and concentrating *in vacuo*. Flash chromatography (applied in CH_2Cl_2; eluted 2% to 3% to 4% MeOH/CH_2Cl_2) afforded the title compound as an off white solid (146 mg, 0.210 mmol, 57%). Mpt: >200 °C; R_f = 0.43 (10% MeOH/CH_2Cl_2); IR (ν_{max}/cm^{-1}, thin film): 3254 (aromatic C-H stretch), 2925 (C-H and N-H stretches), 1706 (C=O stretch), 1589, 1527 (N-H bends), 1405 (S=O asymmetric stretch), 1224, 1140 (C-O and S=O symmetric stretch); ^1H NMR (600 MHz, CD_3CN): δ_H = 2.38 (t, *J* = 7.1 Hz, 2H, **36-H**), 3.27 (ap.q, *J* = 5.6 Hz, 2H, **33-H**), 3.48 (t, *J* = 5.6 Hz, 2H, **32-H**), 3.64-3.67 (m, 4H, **20,37-H**), 4.24-4.26 (m, 2H, **29-H**), 6.58

(bs, 1H, **34-H**), 6.72 (s, 2H, **40-H**), 7.06 (bd, J = 5.4 Hz, 1H, **6-H**), 7.51-7.56 (m, 2H, **15,16-H**), 7.62-7.65 (m, 3H, **5,24-H**), 7.91-7.93 (m, 3H, **14,23-H**), 7.95-7.98 (m, 2H, **12,17-H**), 8.03 (dd, J = 8.5, 1.6 Hz, 1H, **11-H**), 8.20 (s, 1H, **3-H**), 8.31 (bs, 1H, **20-H**), 8.49 (s, 1H, **19-H**); ^{13}C NMR (150 MHz, CD_3CN): δ_C = 33.8 (**C-37**), 33.9 (**C-36**), 38.4 (**C-33**), 64.0 (**C-29**), 68.3 (**C-30**), 68.9 (**C-32**), 110.3 (**C-5**) 114.5 (**C-3**), 115.8 (**C-6**) 117.6 (**C-24**), 123.4 (**C-11**), 124.2 (**C-19**), 126.1 (**C-15**), 126.2 (**C-16**), 127.1 (**C-23**), 127.3 (**C-14**), 127.9 (**C-17**), 128.2 (**C-12**), 129.4 (**C-10**) 132.9 (**C-13**), 133.2 (**C-18**), 133.9 (**C-40**), 135.4 (**C-9**), 135.8 (**C-22**), 145.2 (**C-2**), 142.5 (**C-25**), 144.7 (**C-8**) 153.1 (**C-27**), 169.8 (**C-35**), 170.5 (**C-39**); LRMS m/z (ES^+): 698 $[M+H]^+$; Found 698.2010 $[M+H]^+$; $C_{34}H_{32}N_7O_8S$ requires 698.2033; Anal. Calcd. for $C_{34}H_{31}N_7O_8S$: C, 58.53; H, 4.48; N, 14.05. Found C, 65.36; H, 3.66; N, 12.62%.

3. HPLC Methods

Preparative HPLC

Preparative HPLC machine used with 2 mL injection loop.

Method A: Discovery®BIO Wide Pore C18 (Varian; 100 x 21.2 mm, 5 µm beads) flow rate of 10 mL/min, and UV detection at 215 and 254 nm. Linear gradient: 25-40% B over 8 min (A = H_2O/0.1% TFA, B = MeCN/0.1% TFA).

Method B: Discovery®BIO Wide Pore C18 (Varian; 100 x 21.2 mm, 5 µm beads) flow rate of 10 mL/min, and UV detection at 215 and 254 nm. Linear gradient: 20-30% B over 8 min (A = H_2O/0.1% TFA, B = MeCN/0.1% TFA).

Method C: Discovery®BIO Wide Pore C18 (Varian; 100 x 21.2 mm, 5 µm beads) flow rate of 10 mL/min, and UV detection at 215 and 254 nm. Linear gradient: 25-35% B over 8 min (A = H_2O/0.1% TFA, B = MeCN/0.1% TFA).

Method D: Discovery®BIO Wide Pore C18 (Varian; 100 x 21.2 mm, 5 µm beads) flow rate of 10 mL/min, and UV detection at 215 and 254 nm. Isocratic: 30% B over 20 min (A = H_2O/0.1% TFA, B = MeCN/0.1% TFA).

Method E: Discovery®BIO Wide Pore C18 (Varian; 100 x 21.2 mm, 5 µm beads) flow rate of 10 mL/min, and UV detection at 215 and 254 nm. Isocratic: 35% B over 25 min (A = H_2O/0.1% TFA, B = MeCN/0.1% TFA).

Method F: Discovery®BIO Wide Pore C18 (Varian; 100 x 21.2 mm, 5 µm beads) flow rate of 10 mL/min, and UV detection at 215 and 254 nm. Linear gradient: 32-37% B over 8 min (A = H_2O/0.1% TFA, B = MeCN/0.1% TFA).

Method G: Discovery®BIO Wide Pore C18 (Varian; 100 x 21.2 mm, 5 µm beads) flow rate of 10 mL/min, and UV detection at 215 and 254 nm. Linear gradient: 33-38% B over 15 min (A = H_2O/0.1% TFA, B = MeCN/0.1% TFA).

Method H: Discovery®BIO Wide Pore C18 (Varian; 100 x 21.2 mm, 5 µm beads) flow rate of 10 mL/min, and UV detection at 215 and 254 nm. Linear gradient: 38-42% B over 10 min (A = H_2O/0.1% TFA, B = MeCN/0.1% TFA).

Method I: Discovery®BIO Wide Pore C18 (Varian; 100 x 21.2 mm, 5 µm beads) flow rate of 10 mL/min, and UV detection at 215 and 254 nm. Linear gradient: 2-98% B over 18 min (A = H_2O/0.1% TFA, B = MeCN/0.1% TFA).

Method J: Discovery®BIO Wide Pore C18 (Varian; 100 x 21.2 mm, 5 µm beads) flow rate of 10 mL/min, and UV detection at 215 and 254 nm. Linear gradient: 30-40% B over 8 min (A = H_2O/0.1% TFA, B = MeCN/0.1% TFA).

Method K: Discovery®BIO Wide Pore C18 (Varian; 100 x 21.2 mm, 5 µm beads) flow rate of 10 mL/min, and UV detection at 215 and 254 nm. Linear gradient: 40-50% B over 15 min (A = H_2O/0.1% TFA, B = MeCN/0.1% TFA).

Method L: Onyx Monolithic Semi-Prep C18 (Phenomenex®;100 x 10 mm, 2µm macropore size, 13nm mesopore size) flow rate 10 mL/min; UV detection at 215 and 254 nm. Linear gradient: 25-50% B over 10 min (A = H_2O/0.1% TFA, B = MeCN/0.1% TFA).

Method M: Onyx Monolithic Semi-Prep C18 (Phenomenex®;100 x 10 mm, 2µm macropore size, 13nm mesopore size) flow rate 10 mL/min; UV detection at 215 and 254 nm. Linear gradient: 30-35% B over 10 min (A = H_2O/0.1% TFA, B = MeCN/0.1% TFA).

Analytical HPLC

Method N: Discovery®BIO Wide Pore C18 (Varian; 25 cm x 4.6 mm, 10 µm beads), flow rate 1.0 mL/min, UV detection at 214 nm. Linear gradient: 5-95% B over 20 min (A = H_2O/0.1% TFA, B = MeCN/0.1% TFA).

Method O: Discovery®BIO Wide Pore C18 (Varian; 25 cm x 4.6 mm, 10 µm beads), flow rate 1.0 mL/min, UV detection at 214 nm. Linear gradient: 5-95% B over 30 min (A = H_2O/0.1% TFA, B = MeCN/0.1% TFA).

Method P: Onyx monolithic C18 column (Phenomenex®; 100 x 3.0 mm, 2µm macropore size, 13nm mesopore size), flow rate 1.0 mL/min, UV detection at 214 nm. Linear gradient: 5-95% B over 20 min (A = H_2O/0.1% TFA, B = MeCN/0.1% TFA).

Method Q: Discovery®BIO Wide Pore C18 (Varian; 25 cm x 4.6 mm, 10 µm beads), flow rate 1.0 mL/min, UV detection at 214 nm. Linear gradient: 5-95% B over 20 min (A = H_2O/0.1% TFA, B = MeCN/0.1% TFA).

4. Synthesis of Non-conjugated Peptides

αF-loop WT (**1**)

S A D C L K S C L R M C P D

Standard side-chain protecting groups were used. *Purification*: Method A; *Analysis*: Method N, R_T = 4.81 min; *m/z* (ES+): 798.20 $[M+2H]^{2+}$, 532.14 $[M+3H]^{3+}$, 399.46 $[M+4H]^{4+}$; MALDI TOF^{+}: 1595 (M+)

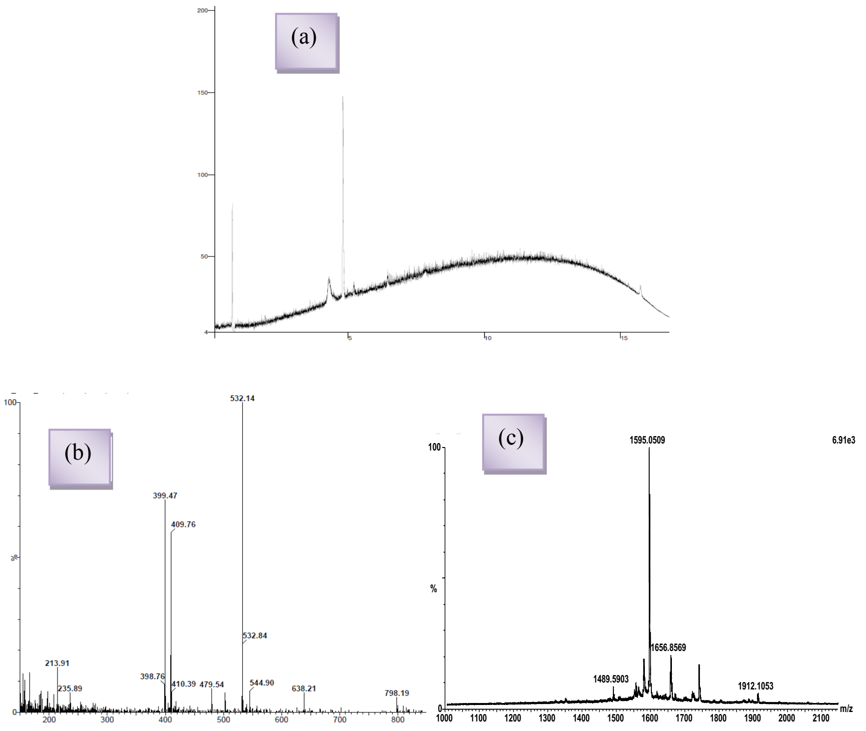

1: (a) Analytical HPLC trace; (b) ES+; (c) MALDI

αF-loop R240C (4)

Field Code Changed

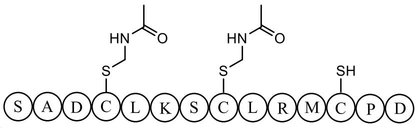

Standard side-chain protecting groups were used with the exception of Fmoc-Cys(Acm)-OH for residues C232 and C236. *Purification*: Method B; *Analysis*: Method N, R_T = 8.41 min; *m*/*z* (ES+): 842.30 $[M+2H]^{2+}$, 561.84 $[M+3H]^{3+}$; MALDI TOF$^+$: 1684 (M+)

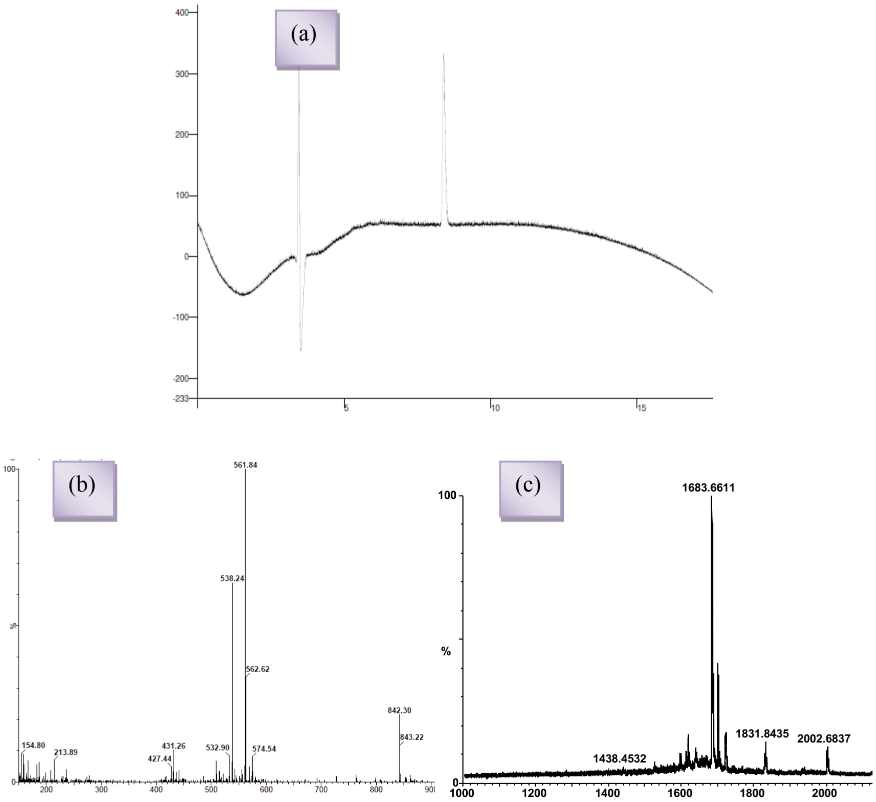

4: (a) Analytical HPLC trace; (b) ES+; (c) MALDI

αF-loop R240SAC (7)

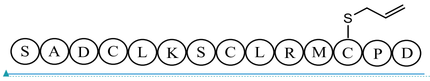

Field Code Changed

Standard side-chain protecting groups were used. S-Allyl Cysteine used as synthesised. *Purification*: Method C; *Analysis*: Method N, R_T = 9.39 min; m/z (ES+): 791.13 $[M+2H]^{2+}$, 527.85 $[M+3H]^{3+}$; MALDI TOF$^+$: 1582 (M+).

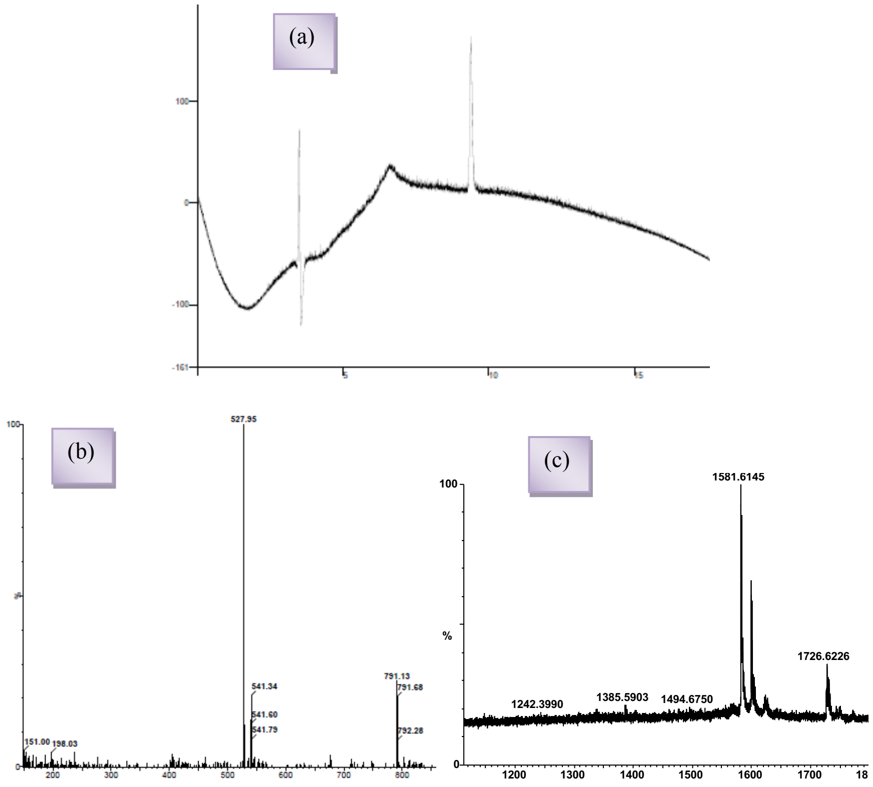

7: (a) Analytical HPLC trace; (b) ES+; (c) MALDI

αF-β10 WT (2)

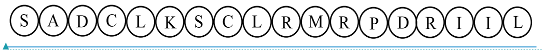

Field Code Changed

Standard side-chain protecting groups were used. *Purification*: Method D; *Analysis*: Method O, R_T = 17.52 min; m/z (ES+): 697.57 $[M+3H]^{3+}$; MALDI TOF$^+$: 2091 (M+).

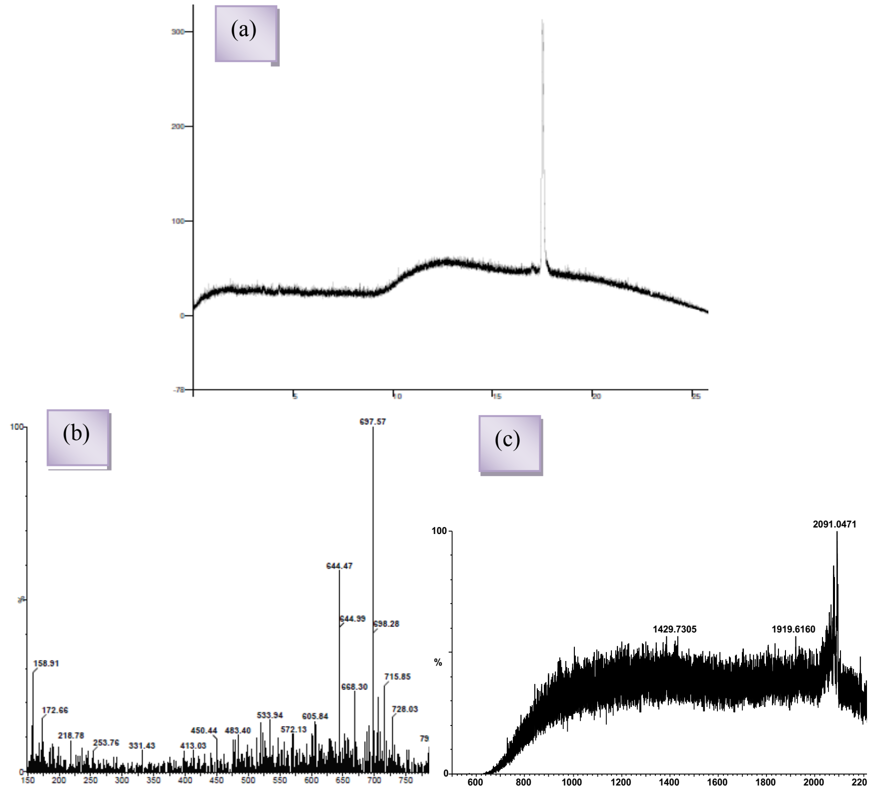

2: (a) Analytical HPLC trace; (b) ES+; (c) MALDI

αF-β10 R240C (**5**)

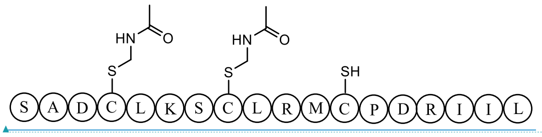

Standard side-chain protecting groups were used with the exception of Fmoc-Cys(Acm)-OH for residues C232 and C236. *Purification*: Method C; *Analysis*: Method N, R_T = 9.60 min; *m*/*z* (ES+): 727.10 $[M+3H]^{3+}$, 545.65 $[M+4H]^{4+}$; MALDI TOF^+: 2180 (M+)

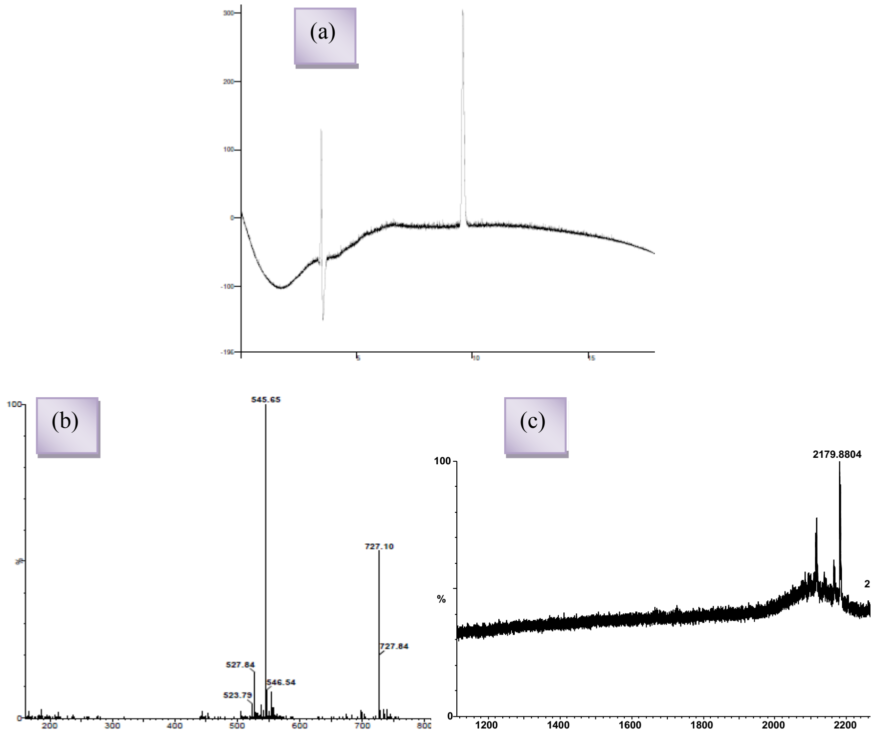

5: (a) Analytical HPLC trace; (b) ES+; (c) MALDI

αF-β10 R240SAC (**8**)

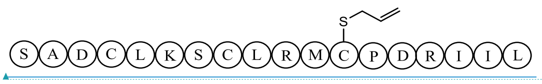

Field Code Changed

Standard side-chain protecting groups were used. S-Allyl Cysteine used as synthesised. *Purification*: Method E; *Analysis*: Method O, R_T = 18.47 min; *m/z* (ES+):693 $[M+3H]^{3+}$.

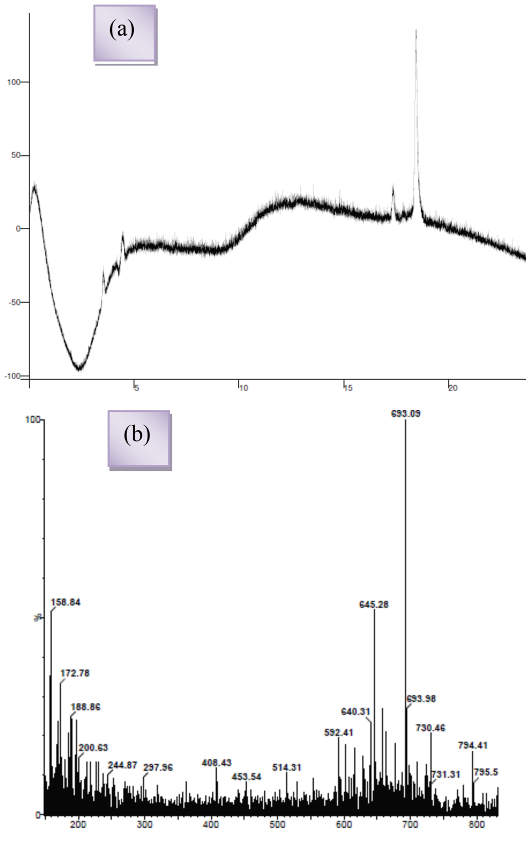

8: (a) Analytical HPLC trace; (b) ES+

B9-αF-β10 WT (3)

Y T Q L F F G G N I T S A D C L K S C L R M R P D R I I L

Field Code Changed

Standard side-chain protecting groups were used. *Purification*: Method F; *Analysis*: Method N, R_T = 10.25 min; m/z (ES+): 1111.75 $[M+3H]^{3+}$, 833.93 $[M+4H]^{4+}$, 667.34 $[M+5H]^{5+}$; MALDI TOF^+: 3332 (M+)

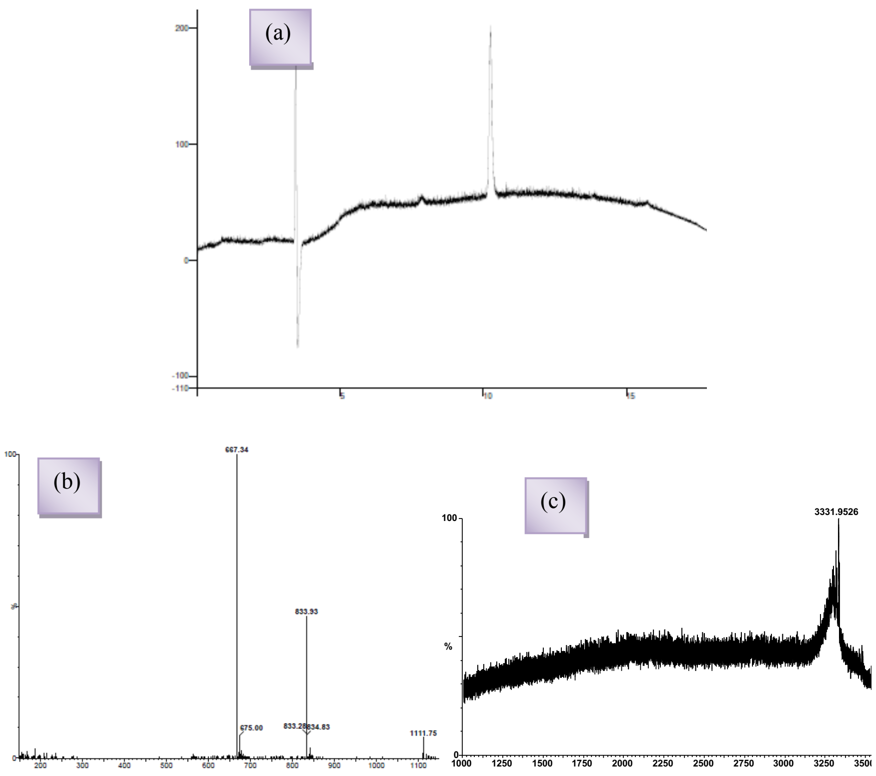

3: (a) Analytical HPLC trace; (b) ES+; (c) MALDI

B9-αF-β10 R240C (**6**)

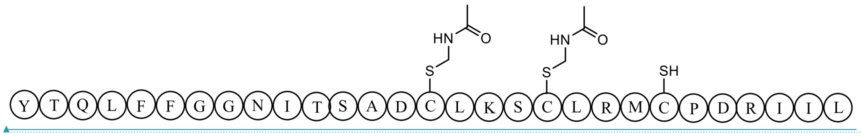

Field Code Changed

Standard side-chain protecting groups were used with the exception of Fmoc-Cys(Acm)-OH for residues C232 and C236. *Purification*: Method G; *Analysis*: Method P, R_T = 6.80 min; *m*/*z* (ES+): 1141.25 $[M+3H]^{3+}$, 856.13 $[M+4H]^{4+}$; MALDI TOF^+: 3421 (M+).

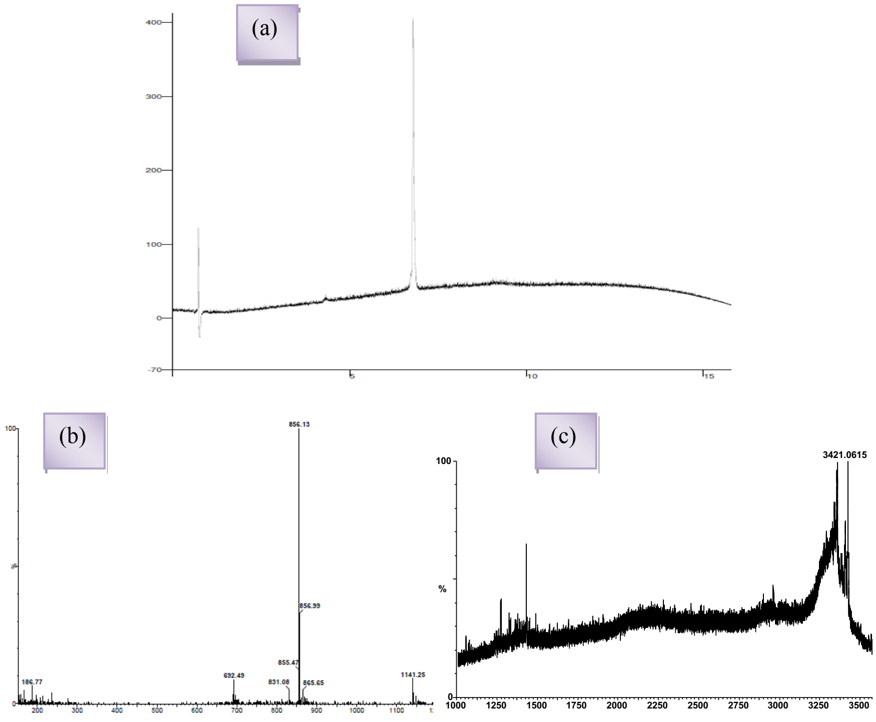

6: (a) Analytical HPLC trace; (b) ES+; (c) MALDI

B9-αF-β10 R240SAC (9)

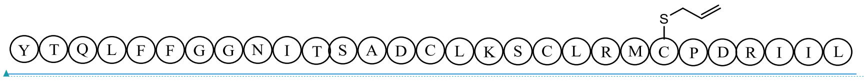

Field Code Changed

Standard side-chain protecting groups were used. S-Allyl Cysteine used as synthesised. *Purification*: Method H; *Analysis*: Method N, R_T = 11.02 min; m/z (ES+): 1107.40 $[M+3H]^{3+}$, 830.52 $[M+4H]^{4+}$; MALDI TOF^{+}: 3319 (M+).

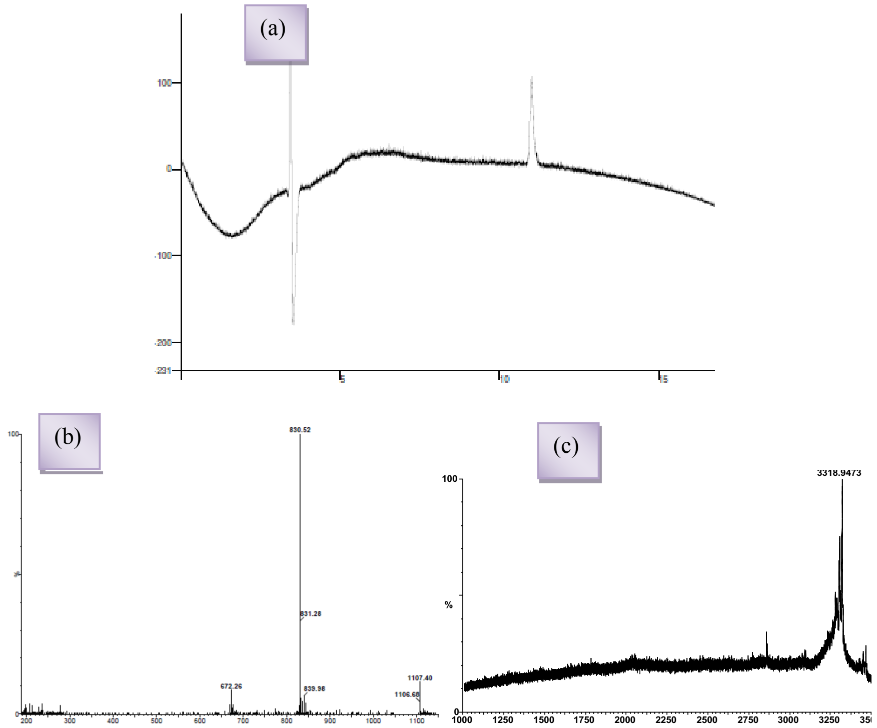

9: (a) Analytical HPLC trace; (b) ES+; (c) MALDI

5. Solution-Based Conjugation

αF-loop (**1** and **17**)

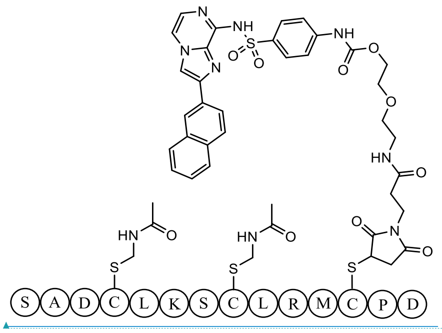

Field Code Changed

Conjugation between **5** and **17** was carried out in solution as described in the Materials and Methods section. *Purification*: Method I; *Analysis*: Method N, R_T = 10.82 min; *m/z* (ES+): 1192.18 $[M+2H]^{2+}$, 794.35 $[M+3H]^{3+}$, 605.63 $[M+K+3H]^{4+}$; MALDI TOF^{+}: 2382 (M+).

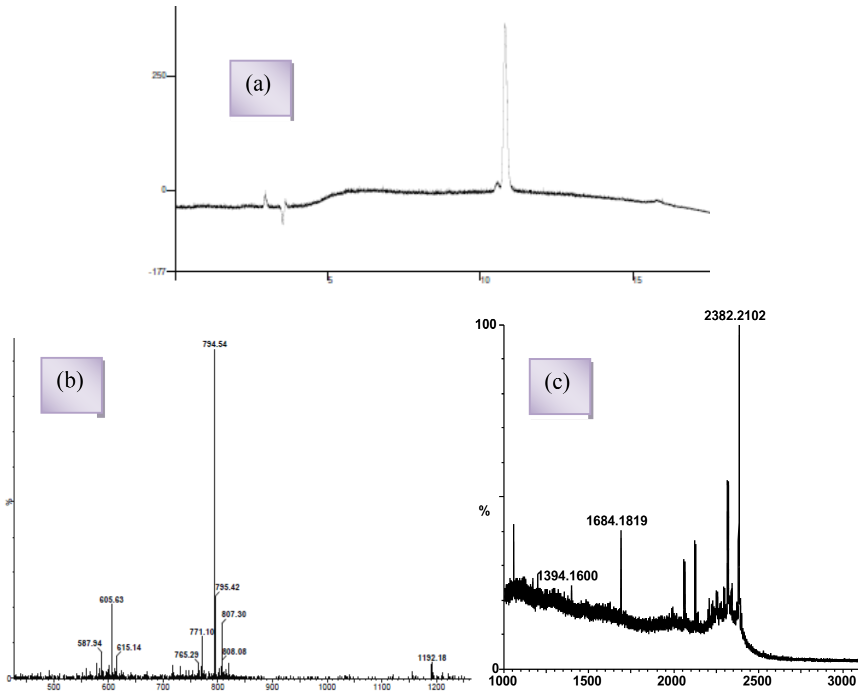

(a) Analytical HPLC trace; (b) ES+; (c) MALDI

αF-β10 (2 and 17)

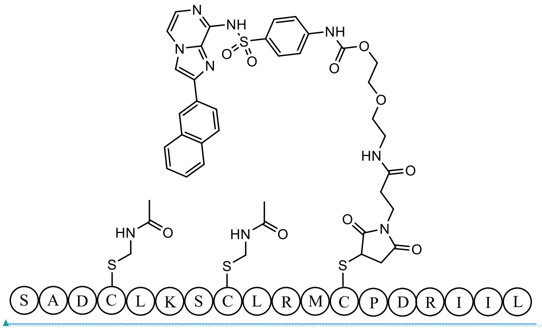

Field Code Changed

Conjugation between **5** and **20** was carried out in solution as described in the Materials and Methods section. *Purification*: Method I; *Analysis*: Method N, R_T = 11.28 min; *m/z* (ES+): 959.86 $[M+3H]^{3+}$, 720.13 $[M+4H]^{4+}$;

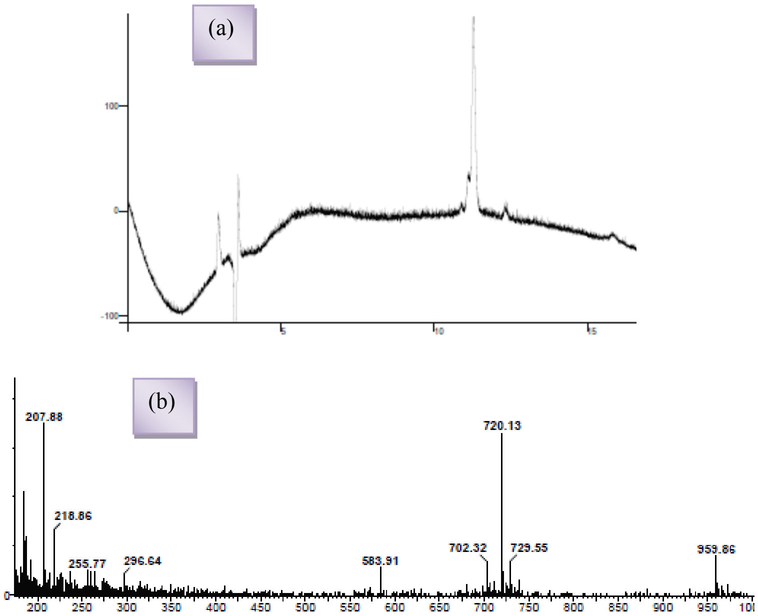

(a) Analytical HPLC trace; (b) ES+

B9-αF-β10 (**3** and **17**)

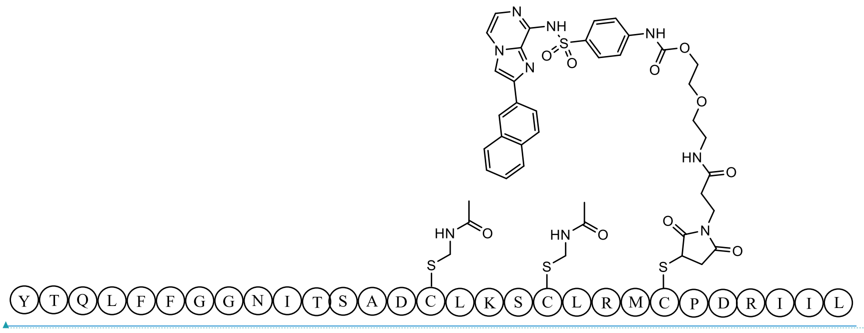

Field Code Changed

Conjugation between **5** and **23** was carried out in solution as described in the Materials and Methods section. *Purification*: Method I; *Analysis*: Method Q, R_T = 5.03 min; *m/z* (ES+): 1374 $[M+3H]^{3+}$, 1031.07 $[M+4H]^{4+}$, 832.29 $[M+K+4H]^{5+}$, 824.35 $[M+5H]^{5+}$, 694.03 $[M+K+5H]^{6+}$.

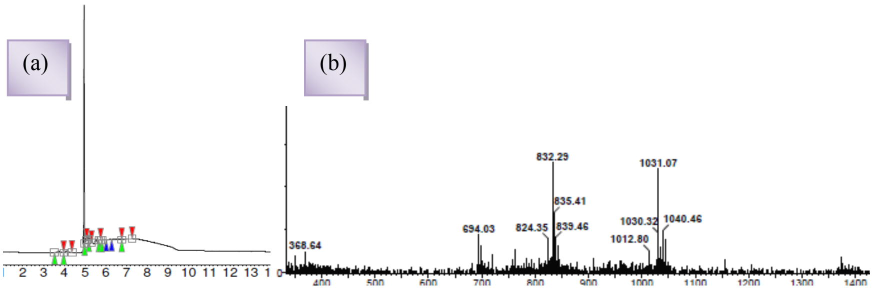

(a) Analytical HPLC trace; (b) ES+

6. Resin-Bound Conjugation

αF-loop (**28**)

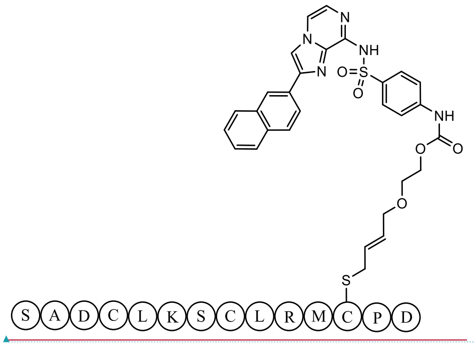

Field Code Changed

Standard side-chain protecting groups were used with the exception of Fmoc-Cys(S^{t}Bu)-OH for residue C240. Conjugation on resin was carried out as described in the Materials and Methods section. *Purification*: Method J; *Analysis*: Method N, R_T = 10.94 min; *m*/*z* (ES+): 1120.62 $[M+2H]^{2+}$, 747.24 $[M+3H]^{3+}$; MALDI TOF^{+}: 2240 (M+)

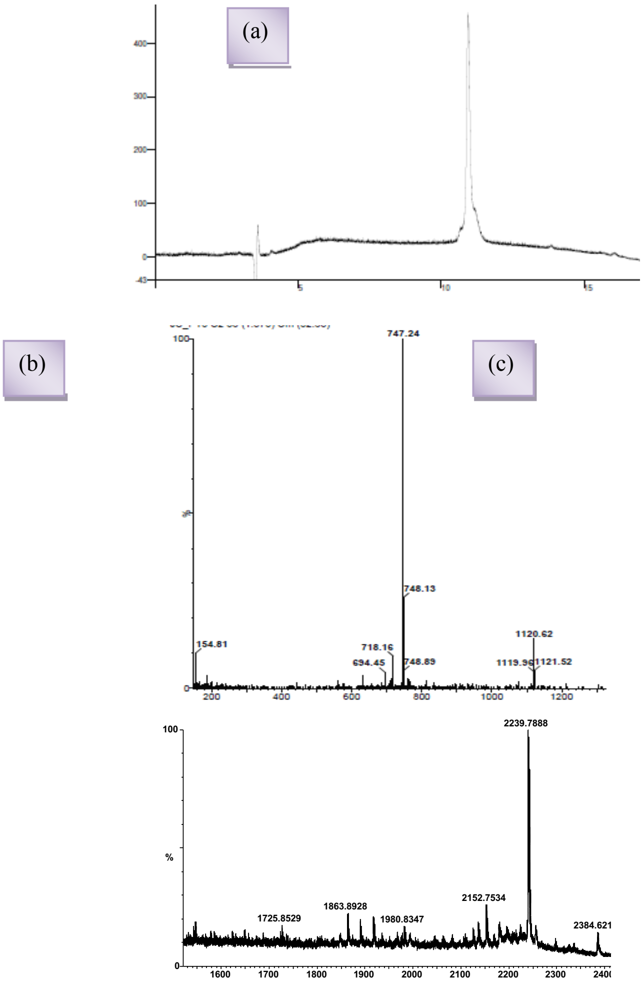

28: (a) Analytical HPLC trace; (b) ES+; (c) MALDI

αF-loop (**29**)

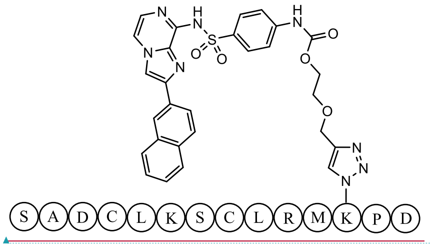

Field Code Changed

Standard side-chain protecting groups were used. Azidolysine was used from available sources within the laboratory. Click Chemistry on resin carried out as described in the Materials and Methods section. *Purification*: Method K followed by Method L; *Analysis*: Method N, R_T = 10.72 min; m/z (ES+): 1067 $[M+2H]^{2+}$, 711.49 $[M+3H]^{3+}$; MALDI TOF$^+$: 2132 (M+)

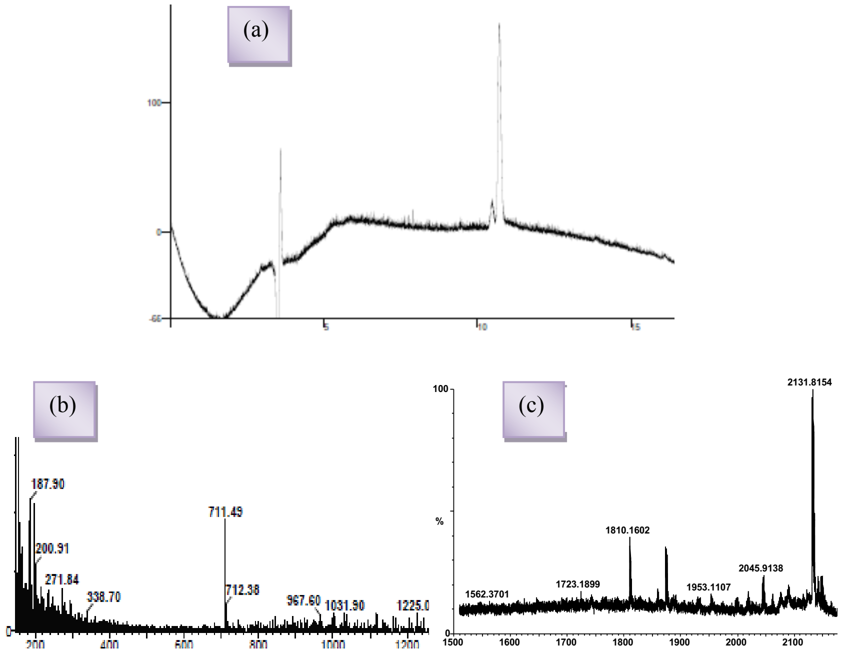

29: (a) Analytical HPLC trace; (b) ES+; (c) MALDI

αF-β10 (30)

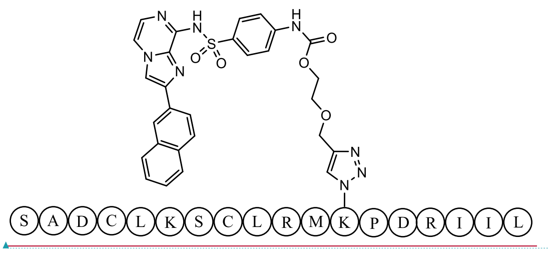

Standard side-chain protecting groups were used. Azidolysine was used from available sources within the laboratory. Click Chemistry on resin carried out as described in the Materials and Methods section. *Purification*: Method H followed by Method M; *Analysis*: Method N, R_T = 11.44 min; *m/z* (ES+): 877.36 $[M+3H]^{3+}$, 847.90 $[M\text{-}(Ser)+3H]^{3+}$, 658.27 $[M+4H]^{4+}$; 636.17 $[M\text{-}(Ser)+4H]^{4+}$.

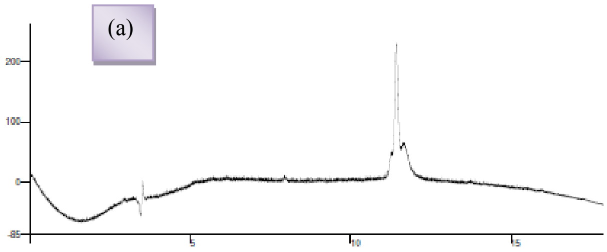

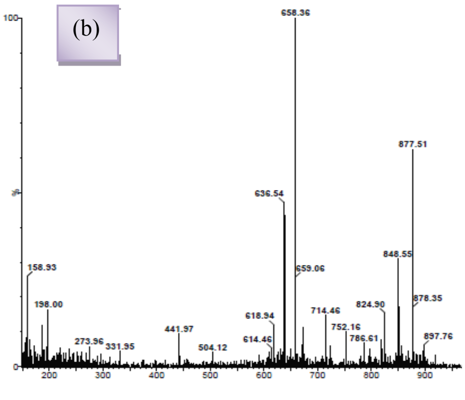

30: (a) Analytical HPLC trace; (b) ES+

Field Code Changed

7. Figure S1

IC50 curves for **14**, **15**, **16**, **27** and **17**. 100% activity corresponds to 100% protein activity.

Compound	IC50 (µM)	95% Confidence interval	
		Error (-)	Error (+)
**14**	88	14	12
**15**	40	17	12
**16**	69	18	14
**27**	124	46	33
**17**	19	5	4

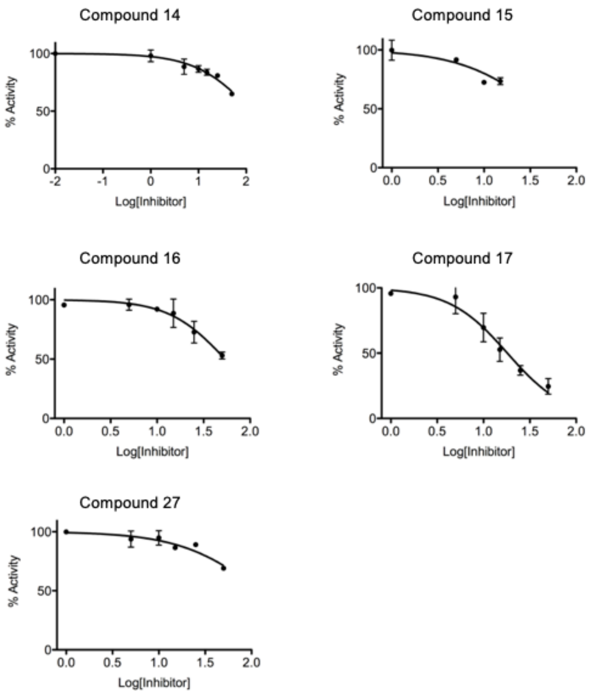

8. Figure S2

(a)

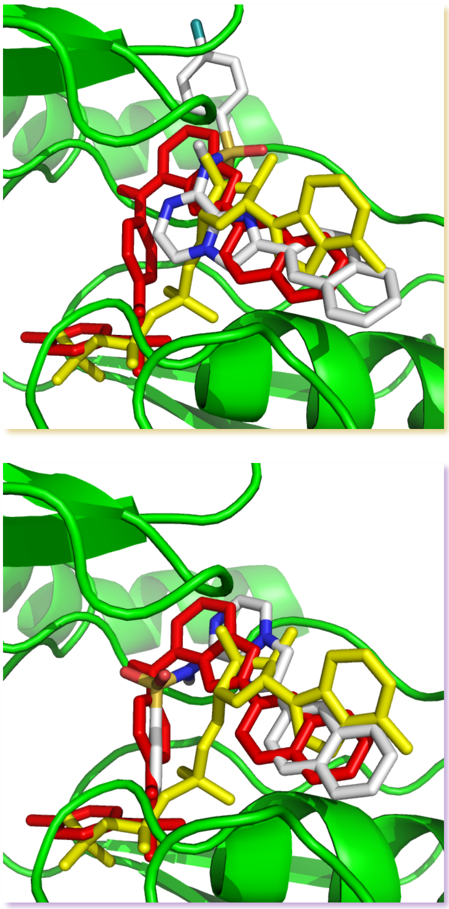

(b)

Figure S21: Overlay of 14 (White, nitrogen atoms indicated in blue) and 13 (Red) with ATPγS (Yellow) in ATPγS-HP0525.

PDB: 1NLY. (a) Lowest energy pose of 14 from cmd (b) 5th lowest energy pose of 14 from cmd. Image generated using PyMOL

9. Figure S3

10 ns simulations of the three peptides were successfully performed on 2 cores; further parallelization on the Legion supercomputer was unsuccessful, most likely due to pressure instabilities resulting from the small system size. Snapshots of the trajectories at different time points and Ramachandran plots show that the longest peptide (ß9-αF-ß10) is the only peptide which is stable during 10 ns. The shortest peptide (αF) is the least stable; it has 2 outliers in the Ramachandran plots even at the beginning of the simulation (after energy minimization).

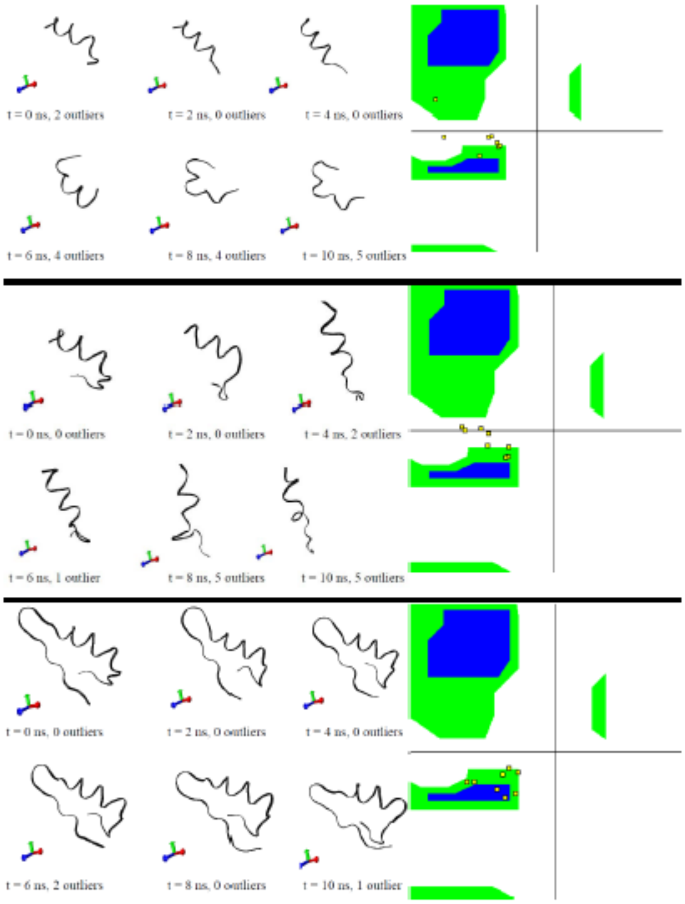

Figure S3: Molecular Dynamics simulations of three different peptides. Top: αF; middle: αF-ß10; bottom: ß9-αF-ß10. Left: Snapshots of the simulation trajectory (every 2ns); the number of outliers in the respective Ramachandran plots is mentioned. Right: Ramachandran plots at t = 10 ns. Only the longest peptide, ß9-αF-ß10 is stable during the entire time. The shortest peptide (αF) is least stable.
